# Root phenotypes for improved nitrogen capture

**DOI:** 10.1007/s11104-023-06301-2

**Published:** 2023-10-04

**Authors:** Jonathan P. Lynch, Tania Galindo-Castañeda, Hannah M. Schneider, Jagdeep Singh Sidhu, Harini Rangarajan, Larry M. York

**Affiliations:** 1 Department of Plant Science, The Pennsylvania State University, University Park, PA 16802 USA; 2 Department of Environmental System Service, ETH Zurich, 8092 Zurich, Switzerland; 3 Department of Plant Sciences, Wageningen University and Research, PO Box 430, 6700AK Wageningen, The Netherlands; 4 Biosciences Division and Center for Bioenergy Innovation, Oak Ridge National Laboratory, Oak Ridge, TN 37830 USA

**Keywords:** Nitrogen, Root, Anatomy, Architecture, Soil, Crop breeding, Root phenotyping, Modeling, Rhizosphere, Plasticity, Physiology

## Abstract

**Background:**

Suboptimal nitrogen availability is a primary constraint for crop production in low-input agroecosystems, while nitrogen fertilization is a primary contributor to the energy, economic, and environmental costs of crop production in high-input agroecosystems. In this article we consider avenues to develop crops with improved nitrogen capture and reduced requirement for nitrogen fertilizer.

**Scope:**

Intraspecific variation for an array of root phenotypes has been associated with improved nitrogen capture in cereal crops, including architectural phenotypes that colocalize root foraging with nitrogen availability in the soil; anatomical phenotypes that reduce the metabolic costs of soil exploration, improve penetration of hard soil, and exploit the rhizosphere; subcellular phenotypes that reduce the nitrogen requirement of plant tissue; molecular phenotypes exhibiting optimized nitrate uptake kinetics; and rhizosphere phenotypes that optimize associations with the rhizosphere microbiome. For each of these topics we provide examples of root phenotypes which merit attention as potential selection targets for crop improvement. Several cross-cutting issues are addressed including the importance of soil hydrology and impedance, phenotypic plasticity, integrated phenotypes, in silico modeling, and breeding strategies using high throughput phenotyping for co-optimization of multiple phenes.

**Conclusions:**

Substantial phenotypic variation exists in crop germplasm for an array of root phenotypes that improve nitrogen capture. Although this topic merits greater research attention than it currently receives, we have adequate understanding and tools to develop crops with improved nitrogen capture. Root phenotypes are underutilized yet attractive breeding targets for the development of the nitrogen efficient crops urgently needed in global agriculture.

## Improved crop nitrogen capture would address several global challenges

Nitrogen is the mineral nutrient required in greatest amounts by plants, yet is rarely available in sufficient quantities to plants that lack symbioses with nitrogen-fixing bacteria. Suboptimal nitrogen availability is therefore a primary limitation to plant growth in terrestrial ecosystems. Before the advent of chemical fertilizers, agroecosystems relied on nitrogen inputs from legumes as rotational or polyculture crops, nitrogen inputs from green manures grown in situ or ex situ, nitrogen inputs from animal manure, and fallowing to accumulate soil organic matter and nitrogen reserves prior to crop production cycles. These practices remain important in modern agriculture and multiple avenues exist to optimize soil management and input use to improve and sustain nitrogen fertility in crop production (Thorup-Kristensen and Kirkegaard 2016; Udvardi et al. 2021).

However, agronomic options to manage nitrogen fertility are limited by a range of factors beyond the scope of this essay, as evidenced by the fact that crop nitrogen nutrition is associated with huge problems in global agriculture. In high-input agroecosystems, intensive nitrogen fertilization is associated with substantial cost, as well as degradation of air and water resources (Woods et al. 2010; Foley et al. 2011). For example, nitrogen fertilizer is the single largest financial cost, energy cost, and environmental cost of maize production in the USA (Northrup et al. 2021). In low-input agroecosystems characteristic of smallholder agriculture in developing nations, suboptimal nitrogen availability is a primary constraint to crop production, and therefore food security, economic development, and political stability (Lynch 2007, 2019; FAO 2015; Nkonya et al. 2016; World Bank 2017). These constraints are intensifying over time because of the synergistic impacts of increasing population pressure, global climate change, and soil degradation (Oldeman 1992; Tebaldi and Lobell 2008; Godfray et al. 2010; St. Clair SB and Lynch 2010; Foley et al. 2011; Lynch 2022a). Agricultural options to mitigate the effects of global climate change through *e.g.* biosequestration and biofuel crops are constrained by the need to manage such systems without the climate-forcing effects of intensive nitrogen fertilization. Global agriculture urgently needs crops and cropping systems capable of sustained productivity with reduced requirement for exogenous nitrogen inputs.

Nitrogen cycling in many agroecosystems is ‘leaky’, releasing significant amounts of nitrogen into surface water, ground water, and the atmosphere. For example, it is estimated that only 25–50% of applied nitrogen fertilizer is taken up by intensive maize monocultures (Hodge et al. 2000; Asghari and Cavagnaro 2011; Modolo et al. 2018). Alongside multiple agronomic options to improve nitrogen inputs from *e.g.* optimizing fertilizer use, soil management, and legumes (Udvardi et al. 2021), improving nitrogen capture by crop roots is a very direct option to sustain production with reduced nitrogen inputs. Crops with greater nitrogen capture would be more productive in systems with limited nitrogen fertilization, notably smallholder agriculture in developing nations and biofuel crops on marginal lands. In high-input systems, such crops would require less nitrogen inputs while reducing nitrogen loss to the environment. As we summarize in this essay, crops display substantial genotypic variation for root phenotypes that are associated with nitrogen capture. Root phenotypes are promising albeit presently underutilized avenues to breed crops with better nitrogen capture.

In this perspective we provide an overview of root phenotypes that are potential targets to improve nitrogen capture by crops. We do not attempt a comprehensive review of a broad and often diffuse literature, but rather highlight promising breeding targets based on the magnitude of potential benefits given natural phenotypic variation present in crops, as evaluated in the field or in realistic controlled environments, and discuss key issues, concepts, knowledge gaps and future prospects. We also focus on studies of specific phenes or basic elements of the root phenotype rather than aggregate traits such as root depth, for reason outlined in Sect. "Phene Integration and Multi-objective Optimization for Breeding Strategies". Many of the results and examples we discuss are drawn from a few crop taxa such as maize, wheat, rice, and common bean. While these taxa represent cereals and legumes, monocots and dicots, they may differ in important ways from globally important taxa that have received less research in this context, such as Brassicaceae, Solanaceae, and root crops.

## Indirect mechanisms to improve nitrogen capture

Multiple root and shoot phenotypes improve nitrogen capture by improving overall plant growth and soil exploration. Vigor, local adaptation, and resistance to stresses all contribute to plant growth generally and via allometric partitioning of biomass between roots and shoots, improve root growth, soil exploration, and nitrogen capture. Phenology is important since it regulates the duration of soil exploration and hence nutrient capture, as well as the duration of nutrient utilization once acquired (Lynch and Rodriguez 1994; Nord and Lynch 2009; Voss-Fels et al. 2018). Phenology is especially important in the context of nitrogen capture since nitrogen availability varies over time. For example, stay-green sorghum genotypes, which have delayed leaf senescence during grain filling, have continued photosynthesis and nitrogen uptake under drought stress while senescent varieties rely on nitrogen and photosynthate translocated from the leaves and other tissues (Borrell and Hammer 2000). In natural ecosystems and low-input agroecosystems, nitrogen mineralization from soil organic matter and vegetation residues is driven by microbial activity that is strongly dependent on seasonal variation in soil moisture and temperature. In high-input agroecosystems nitrogen inputs are generally episodic, with fertilizers often applied early in the crop season. Shoot phenotypes that improve the efficiency of nitrogen utilization are also likely to improve nitrogen capture via improved plant growth. For example, the reduced nitrogen requirement for C_4_ photosynthesis versus C_3_ photosynthesis means that C_4_ plants generate more photosynthate per unit nitrogen invested in leaves, which under limited nitrogen availability would increase both carbon and nitrogen resources for root growth and greater nitrogen capture (York et al. 2022). Root phenotypes that indirectly benefit nitrogen capture include phenotypes that overcome barriers to soil exploration (Lynch and Wojciechowski 2015), the most prevalent being Al toxicity (Delhaize and Ryan 1995), cold soil (Kaspar and Bland 1992), hypoxia (Striker 2012), and mechanical impedance ((Lynch et al. 2022), discussed in Sect. "Anatomical Phenotypes that Improve the Penetration of Hard Soil May Improve Nitrogen Capture"). Many phenotypes that indirectly benefit nitrogen capture are common selection criteria in crop breeding. Indeed, the majority of crop breeding for improved nitrogen capture consists of indirect selection, primarily resistance to biotic stress, vigor, and local adaptation. Such indirect selection is obviously important, but is not likely to be as effective or rapid as would be selection for phenotypes more directly related to nitrogen capture. This is especially true given that most crop breeding occurs with nitrogen fertilization.

## Root architectural phenotypes to improve nitrogen capture

### Spatiotemporal dynamics of soil nitrogen bioavailability

Root system architecture, defined as the physical configuration of the root system, regulates the deployment of roots in the soil in time and space and is therefore a primary determinant of nitrogen capture. Roots are heterotrophic organs that are metabolically costly to build and maintain (Lynch 2014), meaning that root foraging in soil domains with low nitrogen availability, possibly because of nitrogen capture by other roots of the same or neighboring plants, is counterproductive if nitrogen is the limiting soil resource (see Sect. "Low-Input vs. High-Input Systems" regarding multiple resource limitations). To maximize nitrogen capture, root foraging should focus on soil domains with the greatest nitrogen bioavailability, but should only do so to the extent required to exploit that domain. The production and maintenance of more roots than are needed for nitrogen capture in that soil domain will be counterproductive by diverting plant resources from other useful functions, including exploration and exploitation of new soil domains, either directly via the production of new roots or indirectly by *e.g.* greater shoot growth and therefore greater photosynthate production to support further soil exploration.

Mineral nitrogen availability in the soil is spatiotemporally dynamic. Microbial mineralization of soil organic matter occurs in the topsoil, which in most soils, and certainly agricultural soils, has both the greatest concentration of organic matter and favorable conditions for microbial activity. In environments in which water availability and/or soil temperature vary throughout the year, mineralization of soil organic matter can display strong seasonal variation and pulses, as occurs in the spring in temperate systems. Ammonium liberated through mineralization is rapidly converted to nitrate in aerobic soils, which is highly soluble and therefore leaches to deeper soil domains with water. In low-input agroecosystems gradual release of topsoil nitrogen through mineralization combined with rapid nitrogen uptake by plant roots means that nitrogen can be a shallow resource throughout the growing season. When nitrogen fertilizer is used, nitrate, either applied directly or nitrified from ammonium, moves with soil water to deeper soil domains. In low-input agroecosystems mineral nitrogen therefore tends to be a shallow soil resource, whereas when nitrogen fertilizer is used, nitrogen is initially a shallow soil resource but over time becomes a subsoil resource. Therefore, root system architectures that optimize nitrogen capture should enable topsoil foraging, especially in low-input systems, combined with subsequent subsoil foraging, which is important for nitrogen capture in high-input systems and for water capture in all systems (Lynch and Wojciechowski 2015; Dathe et al. 2016). This is a premise of the ‘*Steep, Cheap, and Deep*’ root ideotype for water and nitrogen capture, which integrates architectural, anatomical, and physiological phenotypes (Lynch 2013).

Roots are capable of acquiring dissolved organic nitrogen from the soil solution, a pathway which is especially important in cold soils with slow mineralization such as in alpine and arctic ecosystems (Chapin et al. 1993; Kielland 1994, 1997; Raab et al. 1996, 1999). Organic nitrogen is also important for the nutrition of ectomycorrhizal species such as in temperate forests and heathlands (Smith and Read 2008). Dissolved organic nitrogen can be a significant fraction of total available nitrogen in agricultural soils, especially in soils with high inputs of organic matter and low inputs of mineral nitrogen fertilizers. However, the quantitative importance of dissolved organic nitrogen in crop nutrition is unknown (Gioseffi et al. 2012; Farzadfar et al. 2021). Root phenotypes that may benefit crop nitrogen nutrition by supporting the acquisition of dissolved organic nitrogen are likewise unknown. Dissolved organic nitrogen compounds are generally less mobile than nitrate (Miller and Cramer 2005; Jämtgård et al. 2008) but can represent a significant fraction of leaching loss of nitrogen in agroecosystems (Neff et al. 2003; van Kessel et al. 2009). Several of the phenotypes discussed here employ the paradigm of topsoil foraging for shallow inorganic nitrogen resources such as ammonium and subsoil foraging for leaching resources such as nitrate. This paradigm aligns with results from many field studies, as discussed here and elsewhere in the literature, which may signify that dissolved organic nitrogen is not a major source of crop nitrogen nutrition in most agroecosystems, or could signify that dissolved organic nitrogen is similar to nitrate, and that root phenotypes that improve subsoil exploration improve the capture of both nitrate and dissolved organic nitrogen. This topic merits additional research, but its resolution does not fundamentally alter the concepts presented here.

### Seedling roots

**Fig. 1:**
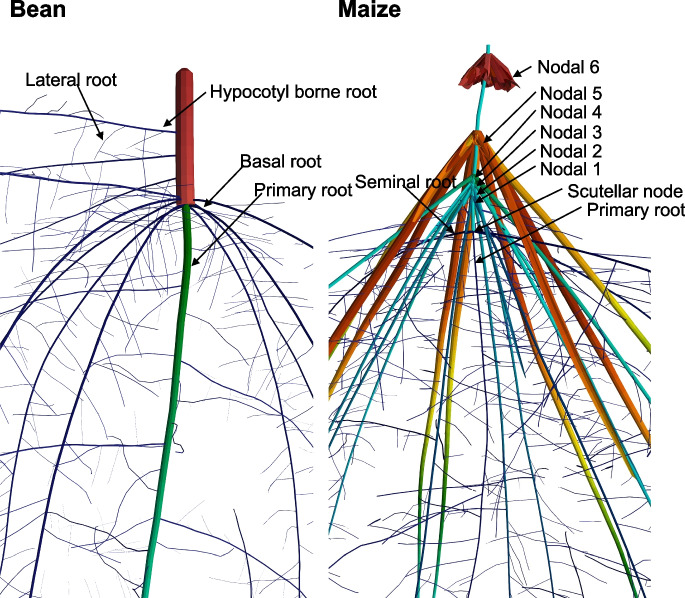
Root classes in a herbaceous dicot crop (common bean) and a herbaceous monocot crop (maize) as visualized in *OpenSimRoot*. Model plants are 40 days post germination, simulations were parameterized from field-grown plants, but with reduced lateral root branching density to aid image clarity. Images and simulations courtesy of Ivan Lopez Valdivia

**Fig. 2:**
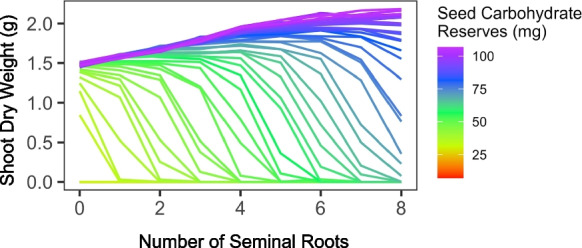
The relationship between seed carbohydrate reserves and optimal seminal root number in a maize landrace growing in a soil with suboptimal nitrogen availability (50 kg ha^−1^ available nitrogen) as simulated in *OpenSimRoot*. Values are means of 20 replicates for each combination of seminal root number and seed carbohydrate reserves. Seed nitrogen and phosphorus content were held constant. Redrawn from Perkins and Lynch 2021

Germinating seeds produce a primary root that descends vertically to assure water capture and plant anchorage (Fig. 1). Cereal crops also immediately extend seminal roots from the base of the mesocotyl with generally shallow growth angles that explore the topsoil. Seminal roots have a smaller diameter than other axial roots and are therefore metabolically efficient, which is important for young seedlings which have limited seed reserves and photosynthate production (Perkins and Lynch 2021). The shallow growth angles of seminal roots are useful for the capture of topsoil nitrogen in seedling establishment and are also complementary to the steeper growth angles of nodal roots that emerge later in development (as shown in maize by Dathe et al. 2016). Multi-objective optimization showed that optimal maize root phenotypes for nitrogen capture have many seminal roots (Rangarajan et al. 2022). In silico analysis estimated that seminal roots account for about a third of nitrogen capture by maize seedlings over the first 25 d of growth, and are especially important in environments with less leaching because of reduced rainfall or heavier soil texture (Perkins and Lynch 2021). Increasing the number of seminal roots should improve nitrogen capture so long as seed carbohydrate reserves could support the increasing root investment (Fig. 2)(Perkins and Lynch 2021). Indeed, this analysis suggested that the varying number of seminal roots among cereal species is driven by seed size, with small-seeded species such as sorghum, rice, pearl millet, and the maize ancestor teosinte unable to support seminal roots at all. In dicotyledonous crops roots emerging from subterranean stem tissue (the hypocotyl in epigeal species, the epicotyl in hypogeal species, (Burridge et al. 2020b)) are functionally analogous to seminal roots in cereals: they are small diameter with shallow growth angles, and are therefore metabolically efficient for topsoil exploration, as has been demonstrated for phosphorus capture (Miller et al. 2003). However, they emerge later than seminal roots, and so may compete with other root classes for photosynthate. For example, optimal common bean root phenotypes for nitrogen capture have few hypocotyl-borne roots, which may reduce intra-plant competition (Rangarajan et al. 2018, 2022). In both maize and bean, optimal seedling root (*i.e.* seminal roots for maize, hypocotyl-borne roots for bean) phenotypes for nitrogen capture have low lateral branching density, since nitrogen is a mobile resource that can be acquired with relatively sparse root length density (Rangarajan et al. 2022).

### Axial roots of mature plants

While the primary root and seedling roots are important for nitrogen capture by seedlings, in mature plants the main axial roots in cereals are the primary root and multiple roots arising from shoot nodes, and in annual dicot species the primary root (or ‘taproot’) and dominant lateral roots arising from it (Fig. 1). In some species like common bean, axial roots emerging from the root/shoot junction are called ‘basal roots’ (Zobel 2011). These axial roots form the structural scaffold from which multiple orders of lateral roots may emerge, and are therefore the primary determinant of root architecture at the organismic scale (Burridge et al. 2020b).

#### Axial root growth angles

**Fig. 3:**
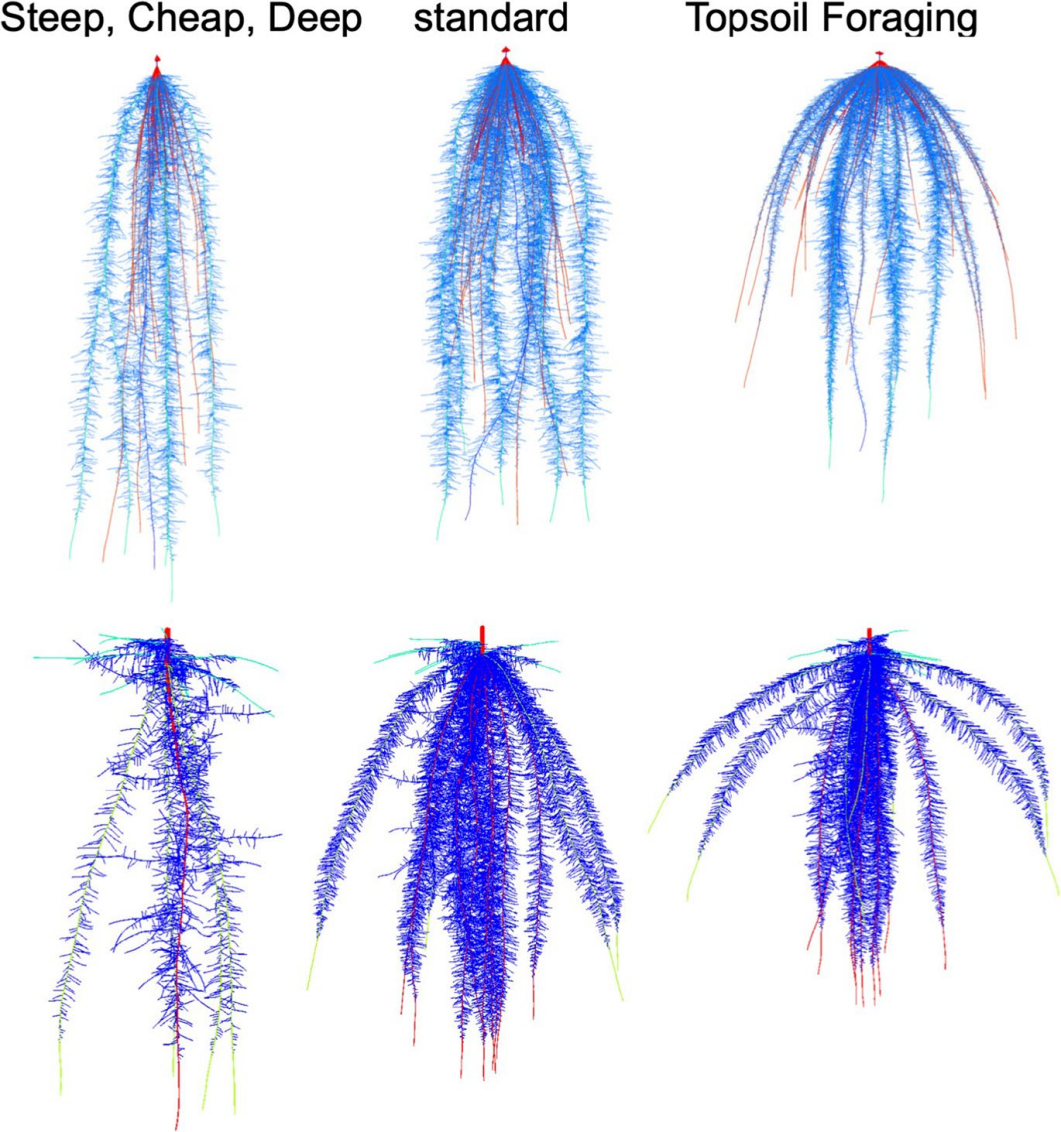
*Steep, Cheap and Deep* and *Topsoil Foraging* ideotypes in maize (top) and common bean (bottom) at 42 days after germination as simulated by *OpenSimRoot*. The center image represents standard phenotypes in maize and common bean germplasm. In maize (representing a nontillering monocot root architecture), the *Steep, Cheap and Deep* phenotype was generated by reducing the number of axial roots, decreasing lateral root branching density, and increasing the steepness of crown root growth angles, while the *Topsoil Foraging* phenotype was generated by doing the opposite. In common bean (representing an annual dicot root architecture), the *Steep, Cheap and Deep* phenotype was generated by reducing the number of hypocotyl-borne roots, reducing the number of basal root whorls, decreasing lateral root branching density, and increasing the steepness of basal root growth angles, while the *Topsoil Foraging* phenotype was generated by doing the opposite. It has been proposed that the *Steep, Cheap, and Deep* phenotype is useful for the capture of subsoil resources including water and leached nitrate, while the *Topsoil Foraging* phenotype is useful for the capture of topsoil resources including recently mineralized nitrate, ammonium, phosphorus, potassium, calcium, magnesium, and in some cases, micronutrient metals. Model parameters are based on empirical observations. Images courtesy of Ernst Schafer. From Lynch (2019)

**Fig. 4:**
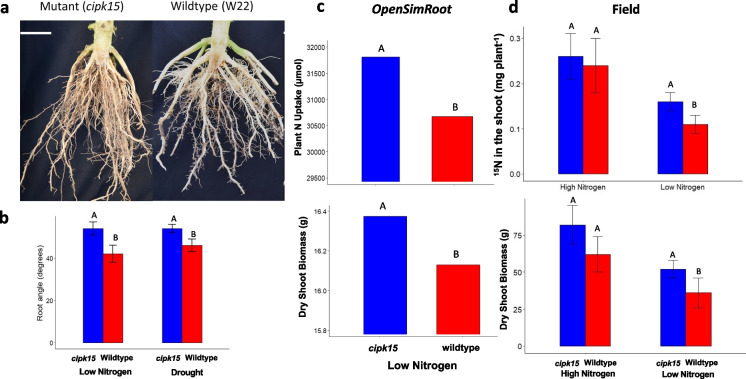
A) Field-excavated root crowns of wildtype and zmCIPK mutant maize genotypes. B) Growth angles (degrees from horizontal) of second node crown roots of wildtype and zmCIPK mutant maize genotypes under water deficit and low nitrogen stress in the greenhouse. C) Nitrogen uptake and biomass of wildtype and zmCIPK mutant maize genotypes from low nitrogen soil at 40 days after germination as simulated in *OpenSimRoot*. D) Nitrogen uptake from deep soil and biomass of wildtype and zmCIPK mutant maize genotypes 70 days after planting in the field in low nitrogen soil. Bars within a panel with different letters are significantly different at p ≤ 0.05. From Schneider et al. (2021a)

The growth angles of axial roots have a strong influence on the rate of descent of roots into deeper soil domains and are therefore important for nitrogen capture (Fig. 3). In several species, genotypic variation in axial root growth angles is associated with rooting depth. In common bean and maize, shallow growth angles enhance topsoil foraging and acquisition of topsoil resources such as phosphorus (Lynch and Brown 2001; Zhu et al. 2005; Lynch 2011, 2022b; Richardson et al. 2011). In common bean, wheat, sorghum and rice, steep growth angles enhance subsoil foraging and water acquisition under terminal drought (Ho et al. 2005; Manschadi et al. 2008; Uga et al. 2011; Mace et al. 2012). Optimal axial root growth angles for nitrogen capture will collocate root foraging with nitrogen availability as it leaches through the soil profile. Growth angles that are too shallow could permit nitrate leaching below the rootzone and are more likely to compete with neighboring plants, especially at high plant densities, whereas angles that are too steep may not adequately exploit the soil volume and would increase competition for nitrogen among roots of the same plant (Lynch 2013). The growth angles of different axial roots and axial root classes should also be complementary with each other to thoroughly exploit available nitrogen while minimizing competition within and among plants (Lynch 2013). These hypotheses were supported by in silico analysis of maize root phenotypes in a range of soil environments, which found that optimal axial root growth angles increased nitrogen capture in a range of environments by 15–50% over 42 d of simulated growth (Dathe et al. 2016). Although extreme root angle phenotypes were beneficial in extreme leaching environments, dimorphic root phenotypes with normal or shallow seminal roots and very steep nodal roots performed well in all scenarios, and consistently outperformed the steep phenotypes (Dathe et al. 2016). Optimization analysis also showed that very steep nodal root growth angles were suboptimal for nitrogen capture, and that in bean, phenotypes with a range of basal root growth angles optimized nitrogen capture through the soil profile more thorough soil exploitation with reduced inter-root competition for nitrogen (Rangarajan et al. 2018, 2022). A study of 108 maize lines in the USA and South Africa found that angles of crown roots (*i.e.* roots emerging from subterranean shoot nodes) were significantly associated with rooting depth, and that most of the best lines in low nitrogen soil had steep angles, either constitutively or in response to nitrogen stress (Trachsel et al. 2013). In maize, a single gene mutation that affects the growth angle of several crown root nodes showed that phenotypes with steep nodal root angles had better nitrogen capture and better plant performance in environments with suboptimal nitrogen availability in the field and in silico (Fig. 4) (Schneider et al. 2022). Axial root growth angle is therefore important for rooting depth and nitrogen capture, and phenotypes optimized for specific production environments are useful breeding targets.

#### Axial root number

**Fig. 5:**
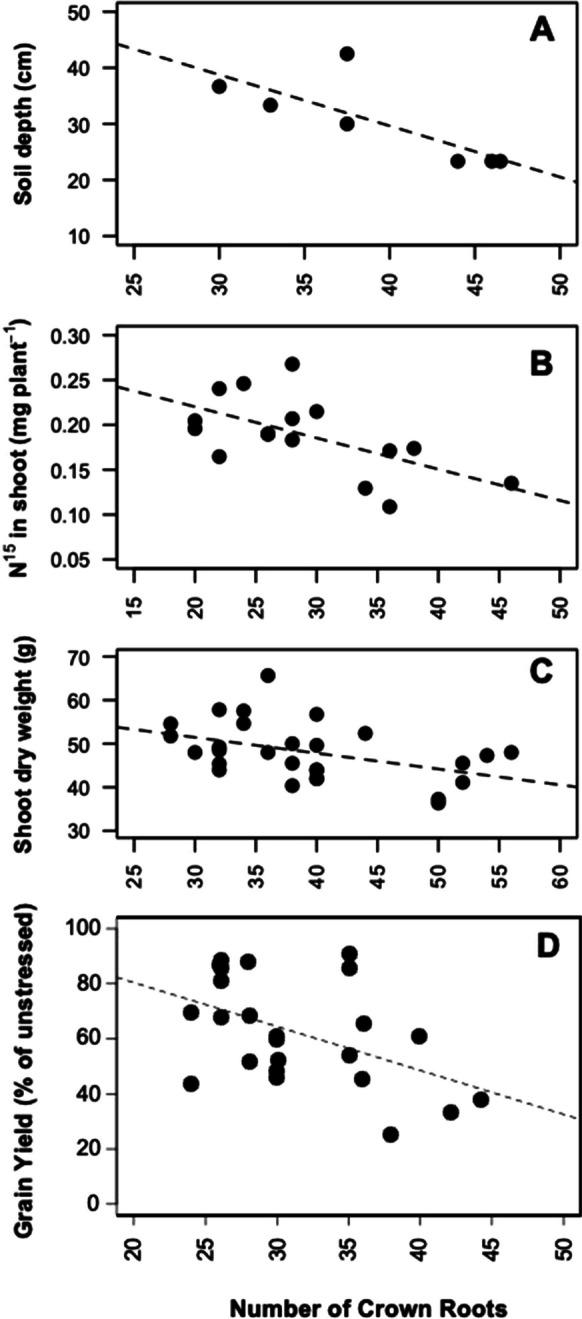
Correlations between crown root number in maize and A) rooting depth (R^2^ = 0.53, p = 0.04), B) ^15^N in shoot (R^2^ = 0.35, p = 0.02), and C) shoot dry weight (R^2^ = 0.16, p = 0.02) at flowering under low nitrogen conditions in the field in South Africa. D) Correlation between crown root number and grain yield (% of yield under high nitrogen) (R^2^ = 0.19, p = 0.02) under low nitrogen conditions in the field in the USA. Redrawn from Saengwilai et al. 2014a

The number of axial roots affects rooting depth and therefore nitrogen capture. The production of a large number of axial roots increases competition within and among plants for nitrogen, and increases competition among roots of the same plant for internal plant resources such as carbohydrates. The production of few axial roots may result in ineffective exploitation of the soil volume, and greater sensitivity to root loss (Sect. "Architectural Phenotypes for Improved Nitrogen Capture Considering Root Loss"). An optimum number of axial roots should therefore exist for nitrogen capture (Lynch 2013). In support of this hypothesis, maize genotypes with fewer nodal roots have deeper rooting, resulting in better capture of deep soil nitrogen, and hence better shoot nitrogen status, photosynthesis, growth and yield under nitrogen stress (Fig. 5) (Saengwilai et al. 2014b). As additional support of this idea, the same pattern is evident under water deficit stress, in which maize genotypes with fewer nodal roots have deeper rooting, better capture of deep soil water, and hence better shoot water status, photosynthesis, growth, and yield (Gao and Lynch 2016). Reduced axial root production in maize increases root depth and water capture under drought in silico (Schäfer et al. 2022a). In contrast, maize genotypes with many nodal roots have shallower rooting depth and greater topsoil exploitation, which in low phosphorus soils results in greater phosphorus capture, leaf phosphorus status, photosynthesis, growth, and yield (Sun et al. 2018). In maize grown in greenhouse mesocosms, reducing the number of nodal roots by physical excision increased root depth, deep nitrogen capture and shoot biomass as reallocation of biomass to lateral and older axial roots increased foraging efficiency (Guo and York 2019). In this context it is interesting that maize grown in aeroponics responds to suboptimal nitrogen availability by reducing the number of crown roots (Gaudin et al. 2011), although this effect may simply result from allometric scaling of root growth with shoot biomass. A reduced nodal root number under nitrogen limitation may be due to either attenuated emergence of nodes producing roots, and/or fewer axial roots per node (York and Lynch 2015; Schneider et al. 2021b). An analysis of US maize cultivars released over the past century showed several root phenotypic changes associated with improved nitrogen capture, including fewer nodal roots in more modern lines (York et al. 2015). In dicots, fewer basal roots and hypocotyl-borne roots increased root depth and increased nitrogen capture (Rangarajan et al. 2018, 2022).

#### Lateral root length and branching density

**Fig. 6:**
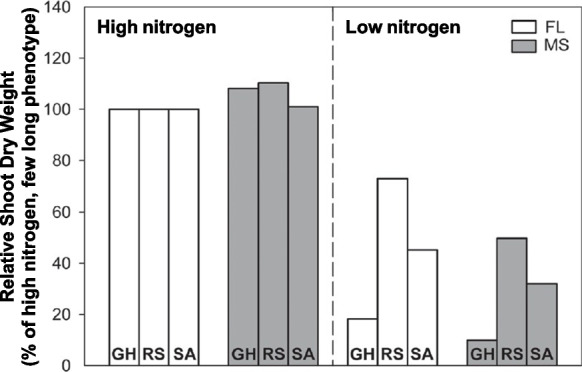
Relative shoot dry weight (% of shoot dry weight in high nitrogen) of maize genotypes with ‘few-long’ (FL) vs. ‘many-short’ (MS) lateral root phenotypes under high and low nitrogen conditions at 42 days after planting in greenhouse mesocosms (GH) and in the field in the USA (RS) and South Africa (SA) at anthesis. From Zhan and Lynch 2015

The rationale for the hypothesis that there exists an optimal number of axial roots for nitrogen capture also pertains to the production of lateral roots (Lynch 2013). Maize root phenotypes with fewer, longer lateral roots (i.e. a ‘few/long’ lateral root phenotype in contrast to a ‘many/short’ lateral root phenotype) had deeper rooting, better nitrogen capture, and better growth in low nitrogen soil in silico (Postma et al. 2014). Under low nitrogen conditions in greenhouse mesocosms and in the field in the USA and South Africa, maize genotypes with a ‘few/long’ lateral root phenotype had deeper rooting and better shoot nitrogen status, photosynthesis, growth and yield (Fig. 6) (Zhan and Lynch 2015). As additional support for this hypothesis, under water deficit in greenhouse mesocosms and the field, maize genotypes with a ‘few/long’ lateral root phenotype had deeper rooting, better capture of deep soil water and hence better shoot water status, growth and yield (Zhan et al. 2015). Reduced lateral root production in maize increases root depth and water capture under drought in silico (Schäfer et al. 2022a). In contrast, maize genotypes with a ‘many/short’ lateral root phenotype have shallower rooting depth and greater topsoil exploitation, which in low phosphorus soils results in greater phosphorus capture, leaf phosphorus status, photosynthesis, growth, and yield (Jia et al. 2018). Similarly, in common bean, a ‘few/long’ lateral root phenotype was beneficial under nitrogen limitation (Rangarajan et al. 2018, 2022).

An analysis of maize genotypes representing 100 years of breeding, corresponding to a period of greater planting density and therefore greater belowground competition for nitrogen, showed that modern lines are more efficient in acquiring nitrogen than older lines because of several changes in root phenotypes, including shallower nodal roots, fewer nodal roots, and reduced lateral branching density, along with anatomical changes (York et al. 2015). That maize breeding has (presumably) inadvertently selected for these architectural elements of the ‘steep, cheap, and deep’ ideotype supports the idea that this phenotype is useful for nitrogen capture.

### Dimorphic architectural phenotypes

As noted above, nitrogen is a shallow as well as a deep soil resource in many agroecosystems. Nitrogen is rarely the single greatest soil resource constraint; in most agroecosystems water, generally a deep resource, is also a constraint, and in many agroecosystems, especially low-input systems and in phosphorus-fixing soils, phosphorus, a shallow resource, is a constraint (Lynch 2019, 2022a, b). For these reasons dimorphic architectural phenotypes capable of both topsoil and subsoil foraging at minimal metabolic cost are useful (Dunbabin et al. 2003; Lynch 2013). Several dimorphic architectural phenotypes are present in annual legume taxa, balancing biomass allocation and growth angle phenotypes among root classes to enable both topsoil and subsoil foraging, although tradeoffs are apparent between allocation to deep and shallow soil foraging (Burridge et al. 2020b). In bean, basal roots emerge from distinct positions or ‘whorls’ that have characteristic growth angles (Burridge et al. 2020b), so that a phenotype with multiple basal root whorls can achieve both shallow and deep foraging. In silico, bean phenotypes with multiple whorls in a fan configuration (i.e. displaying a range of growth angles) performed well under a range of nitrogen regimes including when the topsoil resource phosphorus was limiting (Rangarajan et al. 2018). Optimal root phenotypes for combined nitrogen and phosphorus stress had intermediate architectural phenotypes, and displayed complementary dimorphism through an array of combinations of architectural elements for topsoil and subsoil foraging (Rangarajan et al. 2022). In maize, complementation between shallow seminal roots and steeper nodal roots was advantageous for nitrogen capture (Dathe et al. 2016). Phenotypes with greater lateral root branching density in the topsoil for capture of immobile resources like phosphorus combined with less lateral root branching density in the subsoil for more efficient capture of mobile resources like water and nitrogen would be effectively dimorphic (Postma et al. 2014; Kong et al. 2014).

Strategies to balance topsoil and subsoil exploitation differ between monocotyledonous and dicotyledonous crops, since in monocotyledonous species, the topsoil is explored by continual production of roots from shoot nodes as they descend into the subsoil, in contrast to dicotyledonous species, in which most roots are formed as laterals from existing roots, with the exception of hypocotyl-borne roots, which improve topsoil exploration (Miller et al. 2003). In maize, some genotypes manifest crown roots with progressively steeper growth angles as new nodes emerge (York and Lynch 2015), emphasizing topsoil foraging during seedling establishment, coinciding with the topsoil availability of water, nitrogen, and phosphorus, with progressively deeper soil exploration over time, coinciding with the increasing importance of nitrate and water in deeper soil domains as the season progresses (Lynch 2019, 2022b). In high-input environments, where water remains an important resource limitation but phosphorus and other topsoil resources are abundant, parsimonious root phenotypes that focus on subsoil exploration may be advantageous (Wasson et al. 2012; Lynch 2019, 2022b). However, for most environments root phenotypes that co-optimize topsoil and subsoil foraging at minimal metabolic cost, *i.e.,* without production of so many root axes that yield is adversely affected, should be well adapted. These concepts are implicit in several root ideotypes (Wasson et al. 2012; Schmidt and Gaudin 2017; Lynch 2018, 2019; Burridge et al. 2020b; Uga 2021; Lynch 2022b).

### Architectural phenotypes for improved nitrogen capture considering interplant competition

As noted above, nitrogen capture and plant growth under nitrogen limitation are improved by root architectural phenotypes that reduce competition among root axes of the same plant for internal resources such as photosynthates and assimilated nitrogen as well as for the external resource of bioavailable soil nitrogen. Similar considerations apply to competition among roots of neighboring plants. In an agricultural context, interactions may occur with neighboring plants in monocultures of the same genotype (monogenetic stands), monocultures consisting of different genotypes of the same species (multilines), or polycultures consisting of plants of different species. Interactions of crops with weeds shares concepts with interactions within polycultures with the obvious difference that nitrogen acquired by a weed is generally detrimental to the productivity of the stand or community, whereas nitrogen acquired by one member of a polyculture that is therefore unavailable to another member of the polyculture still contributes to stand performance.

In monoculture, shallow root systems experience greater interplant competition for topsoil resources like phosphorus (Lynch and Brown 2001; Rubio et al. 2001), while steeper root systems experience greater intraplant competition for mobile resources like nitrogen and water (Ge et al. 2000; Nord et al. 2011; Trachsel et al. 2013; Ajmera et al. 2022). Steeper root growth angles increase subsoil exploration but also position root axes of the same plant close together, thereby increasing competition among root axes for soil resources, especially mobile resources such as water (Dathe et al. 2016). Ammonium is a topsoil resource, so shallow architectures of seedling roots, such as caused by shallow growth angles or greater numbers of seminal roots in cereals, may increase interplant competition, although the small size of seedling root systems limits interplant competition. For example, little interplant competition for nitrogen over the first 25 days of maize growth was observed in silico despite varying numbers of seminal roots and plant densities (Perkins and Lynch 2021). It has been proposed that crop breeding for high-input environments with greater plant densities has resulted in indirect selection for root phenotypes adapted to more intense competition for nitrogen (York et al. 2015). Analysis of successful maize varieties over the past century supports this hypothesis, with modern lines having integrated root phenotypes that are better adapted to high density, high nitrogen environments (York et al. 2015). More modern lines had more shallow growth angles, had one less nodal root per whorl, and had double the distance from nodal root emergence to lateral branching, changes which may reduce both interplant and intraplant root competition (York et al. 2015).

Genetic mixtures are common in traditional smallholder agroecosystems. For example, in Africa common bean is often grown in mixtures of up to 15 landraces (Dessert 1987). Compared to monogenetic stands, genetic mixtures have improved yield stability across environments (Smithson and Lenne 1996; Wortmann et al. 1996). It has been proposed that genetic mixtures with contrasting root architecture may improve edaphic stress tolerance by reducing interplant competition and by providing complementary exploitation of distinct soil domains (Henry et al. 2010). This would open the prospect of breeding ‘multilines’ consisting of related genotypes having similar shoot and seed phenotypes but complementary root architecture. A test of this hypothesis with common bean multilines grown in diverse environments in Honduras characterized by both water stress and low soil fertility found instances where specific multilines outperformed their respective monogenetic stands (Henry et al. 2010). However, it was difficult to predict root phenotypes in multilines from those in monogenetic stands because of varying responses to interplant competition.

Polycultures consisting of multiple crops grown together are important in traditional smallholder agroecosystems, and are generally more productive and resilient than their respective monocultures, which is attributed to several mechanisms including complementary nutrient acquisition (Hinsinger et al. 2005; van Ruijven and Berendse 2005; Li et al. 2007). The maize/bean and maize/bean/squash (*i.e.* the ‘3 sisters’) polycultures are historically important and remain so in smallholder systems of Africa and Latin America. These species have contrasting root architectures, and it was hypothesized that in addition to aboveground and dietary synergisms, belowground synergism enabled these polycultures to yield better in low fertility soils (Postma and Lynch 2012). In silico, these polycultures had greater nitrogen capture than their constituent monocultures because of greater niche differentiation (Postma and Lynch 2012). This interpretation was supported by results from field studies showing overyielding of these polycultures in soils with low nitrogen or phosphorus fertility because of niche complementarity (Zhang et al. 2014).

### Architectural phenotypes for improved nitrogen capture considering root loss

Unlike leaves, root axes do not experience programmed senescence, which is probably related to the fact that roots are not terminal organs, and also to the fact that roots are continually lost to adverse soil conditions, pathogens, and herbivores (Norby and Jackson 2000; Fisher et al. 2002). The loss of roots in fertile soil domains can reduce nitrogen capture and results in loss of plant nitrogen in the lost tissue, and also reduces the metabolic costs of sustaining the lost root tissue, which may be beneficial, for example in situations in which too many roots are competing in soil domains with low nitrogen bioavailability.

Root architecture has important effects on root loss by structuring the biotic and abiotic environment of root axes, and has important effects on the consequences of root loss for plant growth, for example by determining the magnitude and utility of the subtending roots lost when an individual root segment is lost (Lynch 2005). The importance of root loss for nitrogen capture is therefore a complex function of several factors including the soil environment, nutrient availability, and root architecture.

**Fig. 7:**
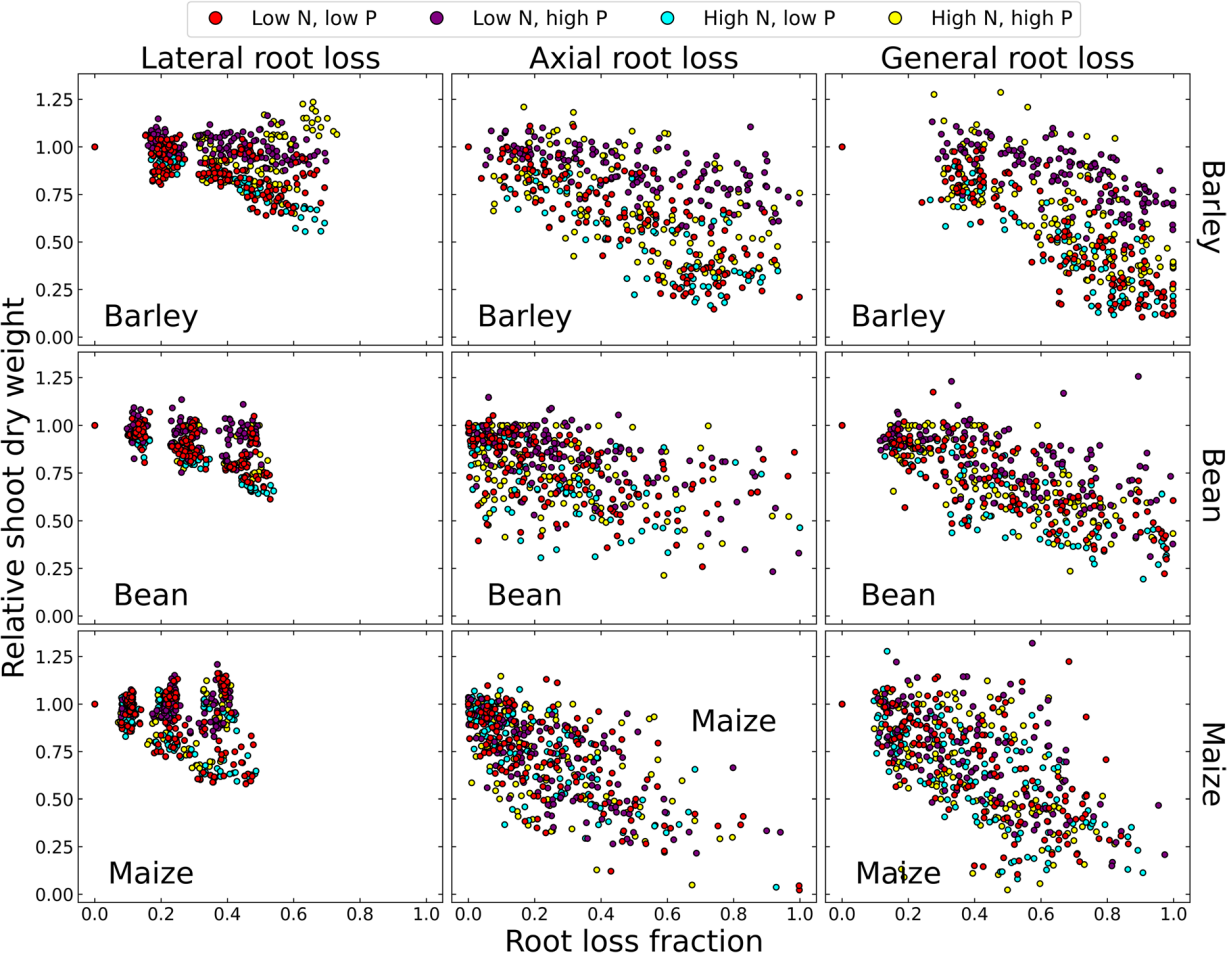
The effects of root loss on shoot dry weight (expressed as a fraction of the shoot dry weight without root loss), for barley, common bean and maize grown under contrasting nitrogen and phosphorus availabilities, as simulated by *OpenSimRoot*. From Schäfer et al. 2022a, b

Given the utility of parsimonious root phenotypes with reduced production of axial and lateral roots for nitrogen capture, as summarized in Sect. "Axial roots of Mature Plants", and the large phenotypic variation for root production evident in crop species, it was proposed that plants produce more roots than are needed for soil resource capture as insurance against inevitable root loss (Lynch 2018). As a corollary of this concept, root ideotypes for high-input environments with some degree of protection from herbivores and pathogens may benefit from more parsimonious root phenotypes, in contrast to plants grown in unmanaged and low-input systems with greater root loss and hence greater need for root proliferation (Lynch 2018). This hypothesis was supported by an *in-silico* study analyzing the effects of root loss in bean, maize, and barley, representing a dicot, a nontillering cereal, and a tillering cereal, respectively, in soil with varying nitrogen and phosphorus availability (Schäfer et al. 2022b). The study found that root loss was more detrimental for phosphorus capture than for nitrogen capture, and indeed that in barley and maize phenotypes with high lateral branching density that were not phosphorus-stressed, loss of lateral roots actually improved plant growth (Fig. 7). Loss of axial roots was detrimental for nitrogen capture however, which is logical since axial roots configure large-scale soil exploration, which is more important than fine-scale foraging in the case of mobile resources like nitrate. Specifically, loss of axial roots reduced the exploration of deep soil domains, which resulted in greater loss of nitrogen to leaching below the effective root zone. While this was an in silico study, results were consistent with empirical studies which however were limited by the difficulty of imposing and assessing specific root loss scenarios (Schäfer et al. 2022b).

In high-input systems with protection from root loss, parsimonious architectures should be useful for improved nitrogen capture, although loss of axial roots may still be detrimental. In low-input systems with greater root loss, phenotypes with a larger number of axial roots may be advantageous for nitrogen capture, although loss of lateral roots does not appear to be detrimental, unless phosphorus availability is suboptimal.

## Root Anatomical Phenotypes to improve nitrogen capture

Root anatomy has many important effects on soil resource capture (Lynch et al. 2021)(Table 1). Here we summarize four categories of effects: 1) effects on nitrogen acquisition near the root surface (*e.g.,* root hairs, Sect. "Long, Dense Root Hairs Improve Nitrogen Capture"); 2) effects on soil exploration by reducing root metabolic costs (Sects. "Anatomical Phenotypes that Reduce the Metabolic Cost of Soil Exploration Improve Nitrogen Capture" and "Subcellular Phenes to Improve Nitrogen Capture"); 3) effects on soil exploration by regulating root responses to mechanical impedance (Sect. "Anatomical Phenotypes that Improve the Penetration of Hard Soil May Improve Nitrogen Capture"); and 4) effects on radial nutrient capture (Sect. "Anatomical Phenotypes that Alter Radial Nutrient Transport May Affect Nitrogen Capture"). Root anatomy also affects nitrogen capture via interactions with soil microorganisms, as summarized in Sect. "Harnessing the rhizosphere microbiome for improved nitrogen capture".

**Table 1:** Root phenes and phene aggregates associated with improved nitrogen capture in annual herbaceous crops, as discussed in the text

class	phene or phene aggregate	phene state	hypothetical mechanism	genotypic variation	example phenotyping platforms	evidence of benefit	notes
Architecture	seedling roots	more seminal roots in cereals	greater topsoil foraging in early vegetative growth	substantial	fast and easy: growth pouches, roll-ups	in silico analysis, few studies	
	hypocoty/epicotyl borne roots in dicots	fewer hypocotyl borne roots in dicots	competition with seminal and lateral roots, needs validation	substantial	shovelomics, soil mesocosms	in silico analysis, few studies, needs validation	potential tradeoff with P capture, sensitivity to seed depth, topsoil moisture
	axial root growth angle	intermediate growth angles are best in most environments, although more extreme phenotypes are suited to more extreme leaching scenarios	deeper rooting/greater concidence of root foraging with N availability	substantial	shovelomics, soil mesocosms	field studies with contrasting genotypes, including with isogenic lines, in silico analysis	in cereals nodal roots emerge over several weeks, and each node may have distinct growth angles, so phenotypes may not manifest in young plants
	axial root number	fewer	reduced intraplant competition/ deeper rooting/greater concidence of root foraging with N availability	substantial	shovelomics, soil mesocosms	field studies with contrasting genotypes, excision studies, in silico analysis	
	lateral root length and branching density	reduced lateral branching density with greater lateral root length	reduced intraplant competition/ deeper rooting/greater concidence of root foraging with N availability	substantial	shovelomics, soil mesocosms	Field and mesocosm studies of contrasting genotypes, in silico analysis	plasticity in some genotypes in response to local resource availability
	dimorphism	balance of topsoil and suboil foraging among axial root classes and nodal positions in cereals	reduced intraplant competition/ greater concidence of root foraging with N availability	substantial	shovelomics, soil mesocosms	Field studies of contrasting genotypes, in silico analysis, few studies	
anatomy	Root hair length and density	Longer, denser hairs	greater soil exploration	substantial	fast and easy: growth pouches, roll-ups	Field and mesocosm studies of contrasting genotypes, in silico analysis	additional benefits for P capture, rhizosheath formation, possible benefits for soil penetration
	Root Cortical Aerenchyma	greater aerenchyma formation	reduced C and N costs of soil exploration by conversion of living tissue to air filled lacunae	substantial	microscopy or LAT of roots excavated from shovelomics, soil mesocosms (Anatomics)	Field and mesocosm studies of contrasting genotypes, in silico analysis	Plastic in response to multiple abiotic factors, useful for water deficit and hypoxia, also affects biotic associations
	Root Cortical Senescence	greater senescence	reduced C and N costs of soil exploration by loss of living cortical tissue	unclear	microscopy or LAT of roots excavated from shovelomics, soil mesocosms (Anatomics), nutrient solution	in silico analysis, few studies	
	Cortical Cell File Number	reduced file number	reduced C and N costs of soil exploration by reducing living cortical area	substantial	microscopy or LAT of roots from seedlings in growth pouches or roll-ups, or excavated from shovelomics, soil mesocosms (Anatomics)	Field and mesocosm studies of contrasting genotypes under water deficit, in silico analysis under N deficit, few studies	
	Cortical Cell Size	greater cortical cell diameter or length	reduced C and N costs of soil exploration by increasing the proportion of vacuolar to cytoplasmic volume in root tissue	substantial	microscopy or LAT of roots from seedlings in growth pouches or roll-ups, or excavated from shovelomics, soil mesocosms (Anatomics)	Field and mesocosm studies of contrasting genotypes under water deficit, in silico analysis under N deficit, few studies	Large cortical cell size is also associated with greater penetration of hard soil
	anatomical adaptations to soil mechanical impedance	eg Multiseriate Cortical Sclerenchyma, root tip shape, cortical structure	greater root depth, improving concidence of root foraging and N availability in leaching environments	substantial for MCS	microscopy or LAT of roots from seedlings in growth pouches or roll-ups, or excavated from shovelomics, soil mesocosms (Anatomics)	in silico analysis, few studies	greater soil penetration by axial roots advantageous for water deficit also
	Secondary Growth	reduced secondary growth in response to N deficit	reduced C and N costs of soil exploration by reducing parenchyma	unknown for N deficit, substantial for P deficit	microscopy or LAT of roots excavated from shovelomics, soil mesocosms (Anatomics)	Field, mesocosm and in silico studies of contrasting genotypes under P deficit, hypothetical under N deficit by same mechanism	
subcellular	cell wall width	greater wall width	reduced symplastic volume in root tissue, reducing maintenance costs, thereby increasing soil exploration	substantial	microscopy or LAT of roots from seedlings in growth pouches or roll-ups, or excavated from shovelomics, soil mesocosms (Anatomics), or possibly leaf tissue if sufficient pleiotropy	Field and mesocosm studies of contrasting genotypes under water deficit, in silico analysis under N deficit, few studies	
	vaculoar occupancy	larger cells (see above)	reduced C and N costs of soil exploration by increasing the proportion of vacuolar to cytoplasmic volume in root tissue	substantial	microscopy or LAT of roots from seedlings in growth pouches or roll-ups, or excavated from shovelomics, soil mesocosms (Anatomics)	Field and mesocosm studies of contrasting genotypes under water deficit, in silico analysis under N deficit, few studies	Large cortical cell size is also associated with greater penetration of hard soil
	mitochondrial density	reduced mitochondrondrial density or activity per cytoplasmic volume	reduced C and N costs of soil exploration	unknown	low throughput/ high accuracy: TEM, confocal microscopy; high throughput /low accuracy: qPCR, mitochondria matrix specific enzyme assay	theoretical	
nitrate transport kinetics	Imax	greater Imax	greater nitrate capture	substantial	ion uptake screening of seedlings in solution culture	in silico analysis, few studies	

### Long, dense root hairs improve nitrogen capture

**Fig. 8:**
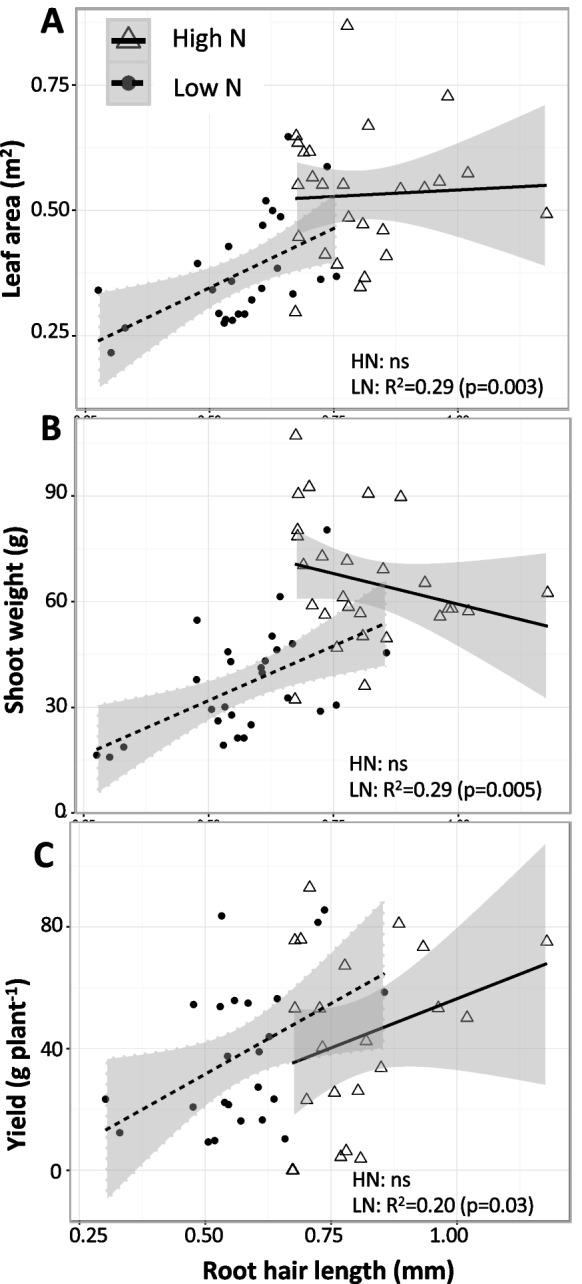
Regression analysis between genotypic variation in root hair length in maize and leaf area (A) and shoot biomass at 63 days after planting (B) and yield (C) under high nitrogen (HN) and low nitrogen (LN) conditions in the field. Gray shading represents 95% confidence interval of the regression line. From Saengwilai et al. 2021

Although the importance of root hairs for the acquisition of nutrients whose mobility in the soil is dominated by diffusion is well known, their value for nitrogen acquisition, which in many soils is driven by mass flow of nitrate in soil water, has been relatively unexplored. Long root hairs may benefit nitrogen capture, particularly when nitrogen diffusion is important. Nitrogen uptake by diffusion is important with low transpiration rates (Phillips et al. 1976), and may be important in deep soil domains, which can provide less transpirational water than shallow soil, as indicated by a field study that used water balance estimates in soil domains of varying depth to conclude that diffusion could contribute up to 85% of the total nitrogen capture (Strebel and Duynisveld 1989). To directly test the hypothesis that long, dense root hairs can improve nitrogen capture, maize genotypes naturally contrasting for root hair phenotypes were evaluated under varying nitrogen regimes in field, greenhouse, and in silico environments (Saengwilai et al. 2021). In all three environments, phenotypic variation in root hair length and density was associated with substantially improved nitrogen capture and plant growth under low nitrogen availability. Notably, in a low nitrogen field environment, genotypes with long root hairs had 267% greater yield than those with short root hairs (Fig. 8). In addition to these direct effects on nitrogen capture, root hairs may have beneficial effects on nitrogen capture by improving penetration of hard soils (Sect. "Anatomical Phenotypes that Improve the Penetration of Hard Soil May Improve Nitrogen Capture") and by improving interactions with rhizosphere communities (Sect. "Rhizosphere Microbial-driven Nitrogen Cycling and Root Anatomy"). Root hair phenotypes deserve greater attention as avenues to improved nitrogen capture in crop breeding.

### Anatomical phenotypes that reduce the metabolic cost of soil exploration improve nitrogen capture

The metabolic cost of soil exploration by roots and their symbionts is significant (Lynch and Ho 2005; Lynch 2014). Anatomical phenotypes that reduce the carbon and nutrient requirements of root growth and maintenance should therefore improve soil resource capture (Lynch et al. 2021). This is especially true of mobile resources like nitrate, which is a deep soil resource in leaching environments.

**Fig. 9:**
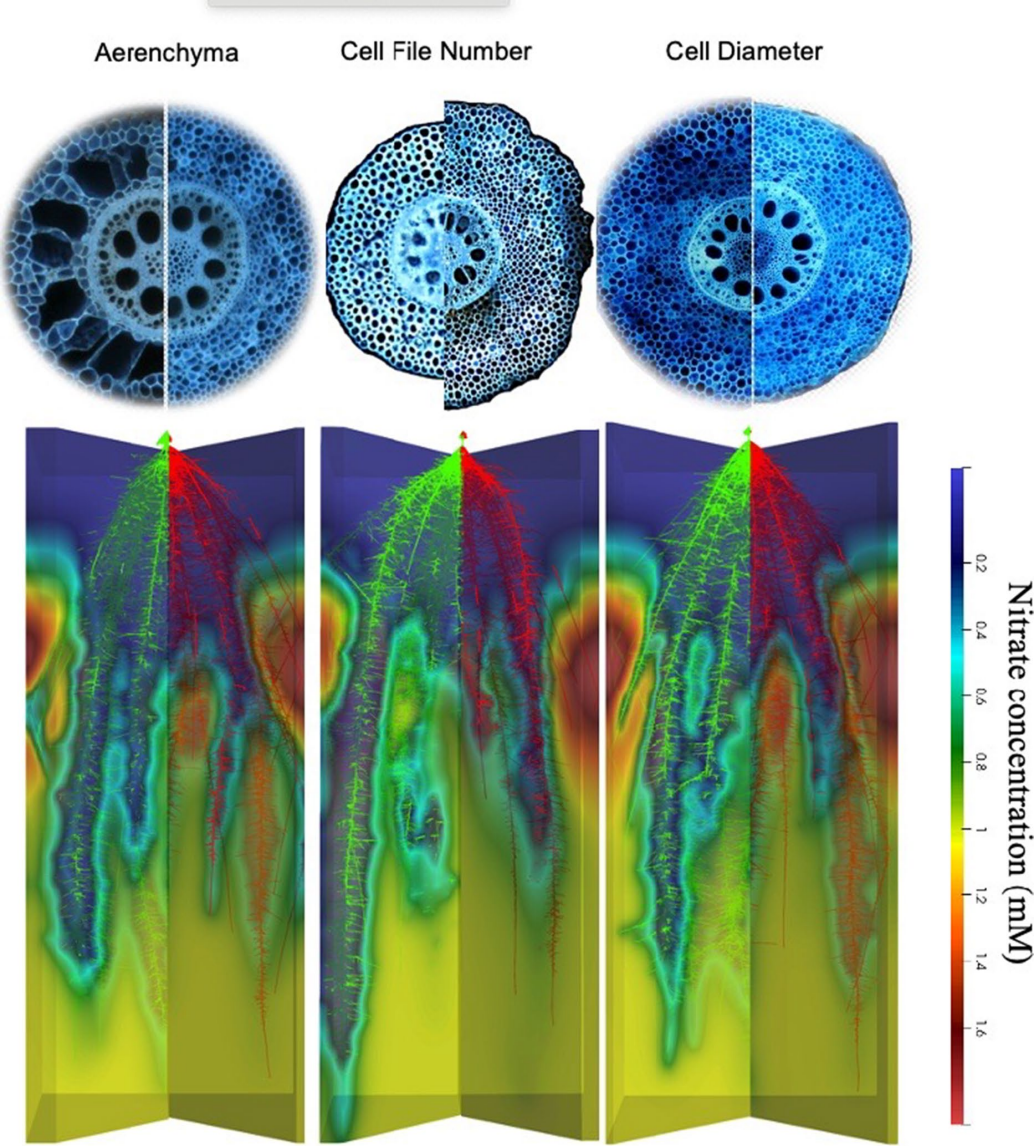
Root cortical anatomy affects growth and nitrate depletion by maize plants in low nitrogen soil as modeled in *OpenSimRoot*. Top panel; phenotypic variation in field-grown maize plants visualized with Laser Ablation Tomography. Bottom panel: Plant growth was simulated for 42 days in a silt-loam soil (soil volume = 25 × 50 × 150 cm) with parameters from field-grown plants. Phene states: cortical aerenchyma (high: 50%, low: 1%), cortical cell file number (many: 16, few: 6), and cortical cell diameter (large: 420 µm^2^, small: 170 µm^2^). Nitrogen availability is 50 kg ha^−1^. Simulations and images courtesy of Ivan Lopez Valdivia

Cortical parenchyma generally comprises a significant portion of primary root tissue, which in monocots persists longer than in dicots, in which the cortex is destroyed in secondary growth (Postma and Lynch 2011b, a; Strock et al. 2018; Strock and Lynch 2020; Lynch et al. 2021). The living cortical area (LCA) of root tissue is well correlated with root respiration, and among contrasting maize phenotypes reduced LCA is associated with greater drought tolerance (Jaramillo et al. 2013). LCA is comprised of several distinct anatomical phenes including root cortical aerenchyma (RCA), root cortical senescence (RCS), cortical cell size (CCS) and cortical cell file number (CCFN) (Lynch 2019; Lynch et al. 2021). Each of these phenes may therefore influence soil exploration and nitrogen capture (Fig. 9).

**Fig. 10:**
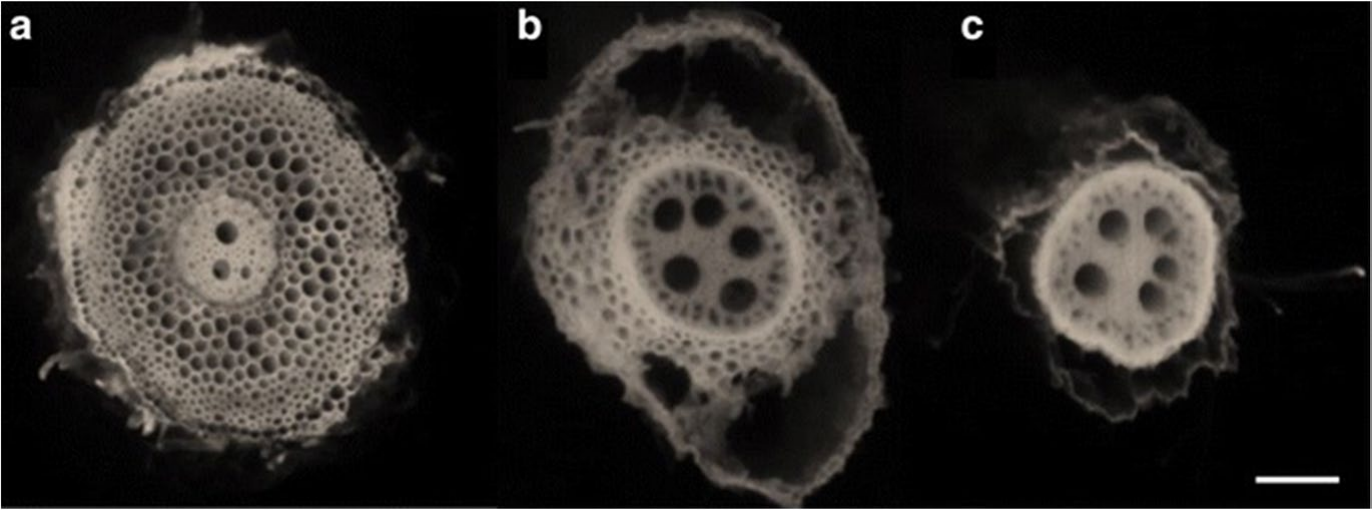
Progression of RCS in a nodal root of barley at 35 days after germination. Root transverse cross sections at A) 4 cm behind the root apex B) 10 cm behind the root apex c) 24 cm behind the root apex. RCS begins in the outer cortical cell files and progresses inwards (B) and eventually all cortical cells will senescence only leaving the stele viable (C). Scale bar = 100 μm. Schneider et al. 2017a, b

Greater RCA formation is associated with substantially greater root growth, soil exploration, nitrogen capture, and plant growth in maize under low nitrogen conditions using the functional-structural plant/soil model *SimRoot* (Fig. 9)(Postma and Lynch 2011a). Growth benefits were ascribed to reduced root respiration as well as nitrogen reallocation from senescing cortical parenchyma to other plant functions during aerenchyma formation. The benefits of RCA for nitrogen capture were greater in coarse-textured soils with greater nitrogen leaching. These in silico results were supported by analysis of maize genotypes contrasting for RCA formation in the field and in greenhouse mesocosms under suboptimal nitrogen availability, where RCA formation was associated with reduced root respiration, greater root depth, greater nitrogen capture, better shoot nitrogen status, and hence greater photosynthesis, growth, and yield (Saengwilai et al. 2014a). Root cortical senescence is similar to RCA but causes entire loss of the cortex in several globally important crops of the *Poaceae*, including wheat, barley, rye, and oat (Fig. 10)(Schneider and Lynch 2018). Loss of cortical parenchyma by RCS reduces root respiration and nutrient content (Schneider et al. 2017b). An in silico study in *SimRoot* showed that RCS is beneficial for barley under suboptimal availability of nitrogen, phosphorus, and potassium driven by reduced root respiration and nutrient reallocation from senescing cortical parenchyma (Schneider et al. 2017a).

An important determinant of the size of the root cortex is the number of cortical cell files formed (CCFN). In maize under drought stress, reduced CCFN was associated with reduced root respiration, greater root depth, better water capture from deep soil, better shoot water status, leaf photosynthesis, growth, and yield (Chimungu et al. 2014b). In silico analysis suggests similar benefits for nitrogen capture under suboptimal availability of nitrogen (Fig. 9) (Lopez-Valdivia et al. 2023).

It has been proposed that variation in cortical cell size (CCS: in this context, cell diameter; cell length is discussed in Sect. "Vacuolar Occupancy") would affect root metabolic cost by two mechanisms: firstly by contributing to LCA and secondly by altering the ratio of cytoplasm to vacuole, since cytoplasm typically has greater nutrient content and respiration than the vacuole. This hypothesis was supported by the observation that in maize under drought stress, greater CCS is associated with reduced root respiration, greater rooting depth, better water capture from deep soil, better shoot water status, leaf photosynthesis, growth, and yield (Chimungu et al. 2014a). In silico analysis suggests similar benefits for nitrogen capture under suboptimal availability of nitrogen (Fig. 9)(Lopez-Valdivia et al. 2023). Larger CCS in wheat grown in compacted soils substantially reduced root respiration and improved penetration of hard soils (Colombi et al. 2019). Penetration of hard soil would benefit nitrogen capture in leaching environments (Strock et al. 2022a; Lynch et al. 2022), (Sect. "Anatomical Phenotypes that Improve the Penetration of Hard Soil May Improve Nitrogen Capture").

The fact that RCA, RCS, reduced CCFN, and reduced CCS all improve the capture of soil resources despite having distinct physiological mechanisms supports the proposal that anatomical phenotypes that reduce the metabolic costs of soil exploration, by reducing nutrient content and respiration, promote greater soil exploration and therefore improve nitrogen capture under low nitrogen availability (Lynch et al. 2021). Comparable benefits were observed in the field, in greenhouse mesocosms with simplified soil biota, and in silico*,* which is a highly simplified environment that serves to test the adequacy of a logic model. It is also noteworthy that these studies examined natural phenotypic variation among well-adapted crop genotypes rather than mutants, which are extreme phenotypes that may suffer from confounding pleiotropic effects.

The majority of the studies cited above were conducted in cereals, which as monocotyledons have a fairly persistent cortex, although RCS can destroy the entire cortex in *Poaceae* (Schneider and Lynch 2018). In contrast, secondary growth in dicotyledonous taxa destroys the cortex, which reduces the importance of root cortical phenotypes for the metabolic costs of mature dicotyledonous root systems. Radial expansion increases the metabolic cost of a root segment simply by adding new tissue, and suppression of secondary growth under edaphic stress may be an adaptive strategy to improve soil exploration (Strock and Lynch 2020). For example, under suboptimal phosphorus availability, common bean genotypes with reduced secondary growth have greater root elongation, increased soil exploration and greater phosphorus acquisition (Strock et al. 2018). It is not known if this occurs in response to nitrogen stress, but it is reasonable to assume that it does. It is also worth noting that the majority of studies relating root anatomy to nitrogen capture by crop plants focus on axial root phenotypes, whereas lateral roots are also responsible for substantial nitrogen capture (Perkins and Lynch 2021).

### Anatomical phenotypes that improve the penetration of hard soil may improve nitrogen capture

Soil mechanical impedance constrains root growth in most soils. Consequent reductions in soil exploration can limit nitrogen capture, especially in subsoils, which are generally hard, and which may contain significant nitrate in arid and semiarid agroecosystems (Lynch and Wojciechowski 2015; Lynch et al. 2022). The importance of root penetration ability for nitrogen capture by maize under varying nitrogen and mechanical impedance regimes was demonstrated in *OpenSimRoot* (Strock et al. 2022a). Soils with plow pans and hard subsoils inhibited root growth but also reduced nitrate leaching, thereby improving the colocalization of bioavailable nitrogen and root length. Improved penetration of axial roots increased rooting depth, thereby increasing nitrogen acquisition and shoot biomass.

**Fig. 11:**
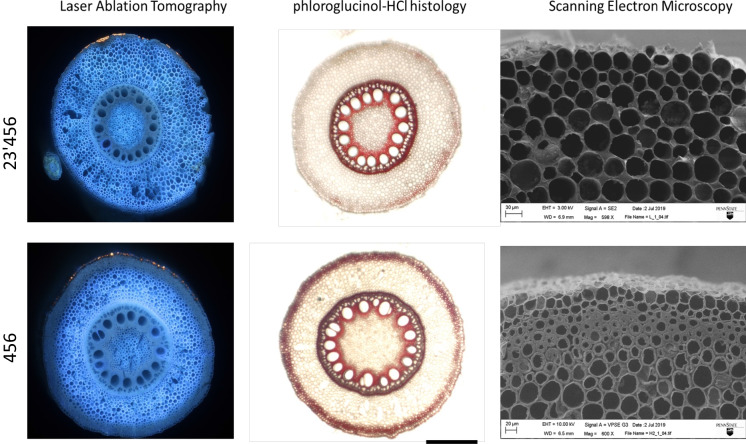
Lines with MCS have smaller and thicker outer cortical cells. These thickened cortical cells are stained red with phloroglucinol–HCl due to their high lignin content. Cryo-SEM images show detailed images of the smaller cells with thick cell walls in the outer cortex (scale bar, 100 μm). Reprinted with permission from Schneider 2022

A number of root anatomical phenotypes have been associated with penetration of hard soils (Lynch and Wojciechowski 2015; Lynch et al. 2021). We will not review them in detail here since root responses to impedance is the subject of considerable literature that seldom focuses on nitrogen capture. Several anatomical phenes have been associated with intraspecific variation for soil penetration, including the shape of the root tip (Colombi et al. 2017), root hair length and density (Haling et al. 2013), cortical cell size (Chimungu et al. 2015; Colombi et al. 2019; Vanhees et al. 2020), and multiseriate cortical sclerenchyma (Fig. 11)(Schneider et al. 2021a). Root hair phenotypes influence the formation of rhizosheaths, which reduce the desiccation and thus the mechanical impedance of soils surrounding root tips (Lynch et al. 2021; Aslam et al. 2022). We propose that these phenes should be explored for their effects on nitrogen capture, especially when they improve the penetrance of axial roots (Strock et al. 2022a; Lynch et al. 2022).

### Anatomical phenotypes that alter radial nutrient transport may affect nitrogen capture

Several root anatomical phenes influence radial nutrient transport, and thereby the spatiotemporal dynamics of nitrogen capture. For example, the progression of RCS in maturing root tissue is correlated with progressively reduced radial hydraulic conductance and radial transport of nutrients including nitrogen and phosphorus (Schneider et al. 2017b). Similarly, RCA formation in maize reduces the radial transport of water and nutrients to the stele (Fan et al. 2007; Hu et al. 2014). Anatomical phenes may reduce radial transport of water and nutrients due to changes in apoplastic and cell-to-cell pathways, reduced contact between the root and soil, and increased endodermal suberization, which occurs during RCS formation. The development of both RCS and RCA presumably disrupts the continuity of the apoplastic and symplastic pathways resulting in reduced cross-sectional area for transport across these pathways and a longer path length (Schneider et al. 2017b). However, reduced radial transport of water and nutrients caused by both RCA and RCS in older root tissue may not be detrimental to plant fitness because the majority of resource capture occurs in younger root segments and lateral roots, which typically do not form RCS and RCA (Schneider and Lynch 2018; Lynch et al. 2021). The development of RCA and RCS in axial root tissue may have little effect on total plant nitrogen uptake because these phenes typically develop in older root segments that occupy soil domains that have already been depleted of nutrients by the younger root tissue of the same or adjacent root axes. However, understanding the spatiotemporal pattern of RCS and RCA development in the root system and collocating root foraging and nitrogen availability as it leaches through the soil profile is an important consideration for the functional utility of these phenotypes for nitrogen capture.

## Subcellular phenes to improve nitrogen capture

Subcellular organelles play important direct and indirect roles in nitrogen metabolism (Britto and Kronzucker 2001; Martinoia et al. 2007). Interplay among subcellular organelles including mitochondria, chloroplasts, vacuoles, and Golgi vesicles ensures the homeostasis of nitrogen and the balance among different pools of nitrogen within a plant cell (Feng et al. 2020). Once acquired, either as ammonium, nitrate, or organic forms, nitrogen is partitioned into proteins, nucleic acids, amino acids, nitrate, and secondary compounds. Among these, protein nitrogen is the largest pool (80%) followed by nucleic acids (5%), amino acids (5%), low molecular weight organic compounds (5%), and soluble nitrogen pools (5%) including ammonium and nitrate (Brown and Cartwright 1953; Wagner et al. 1981; Belton et al. 1985; Close and Beadle 2004). Subcellular organelles, including cytosolic and vacuolar compartments, can define the nitrogen content of each pool and total nitrogen content per cell, which in turn dictates the nitrogen content of root tissue. The vacuole has a much lower concentration of nitrogen compared to the cytosol (Brown and Cartwright 1953; Belton et al. 1985). Therefore, an increase in the ratio of vacuolar to cytoplasmic volume in a tissue would reduce tissue nitrogen content (Lynch 2015). Subcellular organelles can also alter root maintenance and construction costs, important aspects for an efficient nitrogen capture considering the heterotrophic nature of roots (Sect. "Anatomical Phenotypes that Reduce the Metabolic Cost of Soil Exploration Improve Nitrogen Capture"). Several subcellular phenotypes could potentially influence the nitrogen demand of root tissue, which would in turn influence the nitrogen cost of soil exploration and further nitrogen capture, but research on this topic is scarce. Subcellular phenotypes and their interaction with anatomical and architectural phenotypes could represent a new suit of breeding targets for improved nitrogen capture.

### Cell wall thickness

**Fig. 12:**
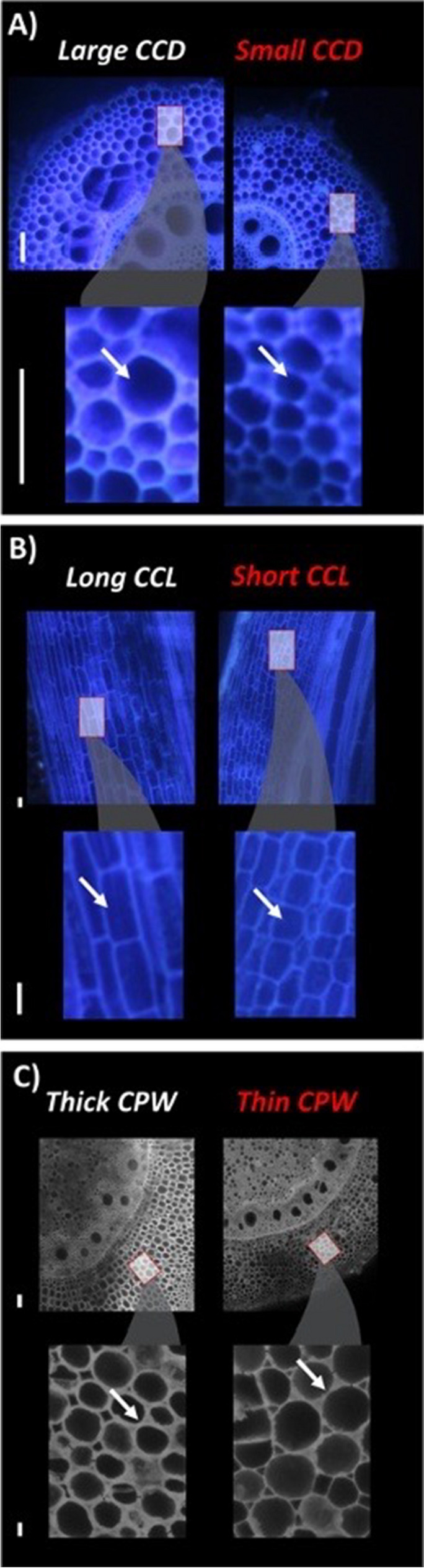
Subcellular phenes regulate the metabolic cost of soil exploration. All images were acquired using Laser Ablation Tomography from maize roots (node 4 (A, C) and node 2 (B) axial roots, 5 cm from base) from plants grown in greenhouse mesocosms (A, B) or the field (C). Phene states in red have a greater metabolic cost than their counterparts. An increase in cortical cell diameter (CCD, A) and cortical cell length (CCL, B), driven by vacuole size, reduces the metabolic cost of root exploration. Similarly, a decrease in cortical cytoplasmic volume due to an increase in cortical parenchyma wall width (CPW, C) also reduces root metabolic costs. The white arrow in each inset points to a representative phene state for the respective phenotype. The scale is 100 µm

Parenchyma cell wall thickness can regulate root metabolic cost and may improve plant performance under suboptimal nitrogen availability (Fig. 12)(Lynch et al. 2021). Significant genotypic variation in cell wall thickness in cortical parenchyma cells has been reported (Flexas et al. 2021). Since the cortex occupies a significant portion of the volume of primary root tissue (which is reduced by RCA and RCS in monocotyledonous roots and by secondary growth in dicotyledonous roots), alterations in the cell wall: cell lumen ratio would have an impact on root metabolic cost. Biosynthesis of secondary cell walls entails construction costs, but the maintenance cost of the cell wall is relatively low compared to the cell lumen (Hamann and Denness 2011; Mahmoudabadi et al. 2019; Shameer et al. 2020). Therefore, root cortical cells with an increased cell wall: cell lumen ratio could lead to reduced tissue root metabolic cost. We hypothesize that genotypes with increased thickness of root cortical cell walls would perform better under nitrogen limitation because of reduced tissue nitrogen content and associated metabolic costs. Another potential benefit of thicker cell walls would be an increase in root tensile strength, which increases penetration of hard soil (Schneider et al. 2021a). Soil hardness increases with depth in most soils, which can hinder nitrogen capture from deep soil (Sect. "Root Anatomical Phenotypes to improve nitrogen capture"c), therefore, if thicker cortical cell walls improve penetration of hard soil, they may also improve nitrogen capture from deep soil. Cortical cell wall thickness is a novel phene that merits attention as an avenue to improve nitrogen capture and use efficiency.

### Vacuolar occupancy

The cytosol contains energy-demanding entities and functions including mitochondria, ribosomes, the endomembrane system, maintenance of transmembrane electrochemical gradients, and turnover of proteins and nucleic acids (Solymosi and Schoefs 2019). In contrast, the vacuole has little energy and nitrogen demand (Brown and Cartwright 1953; Dünser et al. 2019). Tissue with greater proportion of cytosolic to vacuolar volumes generally have higher metabolic rates. For example, root cortical cells in the meristematic zone with many but small vacuoles have greater respiration and nutrient content compared to mature root cortical cells with larger vacuoles (Dünser et al. 2019). At a tissue scale, the ratio of vacuolar to cytoplasmic volume is also influenced by cell size (Sidhu et al. 2023). In larger cells, the ratio of vacuolar to cytoplasmic volume increases. Since the vacuole has both lower metabolic cost and reduced nitrogen content, root phenotypes with larger cortical cells may have improved nitrogen economy.

Cell size can be altered by changes in cell diameter or cell length (Fig. 12). As discussed in Sect. "Anatomical Phenotypes that Reduce the Metabolic Cost of Soil Exploration Improve Nitrogen Capture", larger cortical cell diameter improves maize growth under drought stress (Chimungu et al. 2014a), and low nitrogen availability (Lopez-Valdivia et al. 2023). Larger cortical diameter in wheat reduces root respiration and improves penetration of hard soil (Colombi et al. 2019). Unlike cortical cell diameter, cortical cell length is relatively unexplored, especially in relation to its potential for nitrogen capture and use. We propose that increased cortical cell length can improve nitrogen capture by increasing root elongation rate, and improve nitrogen capture and use efficiency by reducing root metabolic cost and tissue nitrogen content.

For both the diameter and length of root cortical cells, significant natural variation exists in major cultivated crops (Fig. 12)(Chimungu et al. 2015; Colombi et al. 2019; Wang et al. 2013). Therefore, both cortical cell diameter and cortical cell length merit attention for their potential utility in improving nitrogen capture and use efficiency.

### Mitochondrial density

Like all other eukaryotes, plants can manipulate mitochondria directly to regulate metabolic processes including respiration (Millar et al. 2010). For example, plants can reduce the mitochondrial density (*i.e.*, number of mitochondria per unit symplastic volume) as the cells transition from a meristematic phase to maturity, or mitochondrial density is adjusted depending on cell function, for example xylem parenchyma and phloem companion cells tend to have greater mitochondrial mass to support the energy-intensive process of transporting solutes (Steudle and Peterson 1998; Cayla et al. 2015). Differences in mitochondrial density among different cell types are well documented, however, less is known regarding genetic variation in mitochondrial density within the same tissue (such as the root cortex). We hypothesize that genotypic variation in mitochondrial density would provide avenues to select genotypes with lower mitochondrial load in root cortical cells. Reduced mitochondrial density may reduce root respiration and tissue nitrogen content, and hence may be a selection criterion for metabolically cheap roots. As highlighted in Sect. "Anatomical Phenotypes that Reduce the Metabolic Cost of Soil Exploration Improve Nitrogen Capture", "Cell Wall Thickness", and "Vacuolar Occupancy", cheap roots would be beneficial for improving nitrogen capture and use efficiency.

## Nitrogen uptake kinetics: scaling from transporters to the root system

Root system architecture largely governs *where* roots are in the soil and *how many* roots are foraging in a given area, but another primary consideration for nitrogen uptake is what the roots are *doing*. Uptake kinetics refers to both the ability of roots to take up nutrients at low concentrations, as well as the maximum uptake rate (i.e., I_max_ or V_max_) at high concentrations, usually expressed on a *per mass* or *per length *basis (Griffiths and York 2020). In the case of nitrate, several transporters have been discovered that are encoded in the genome and expressed as trans-membrane proteins. These transporters typically have a substrate binding site and the ability to reconfigure their 3D structure in order to allow nutrients to pass from the external soil solution to inside the cells of the epidermis.

**Fig. 13:**
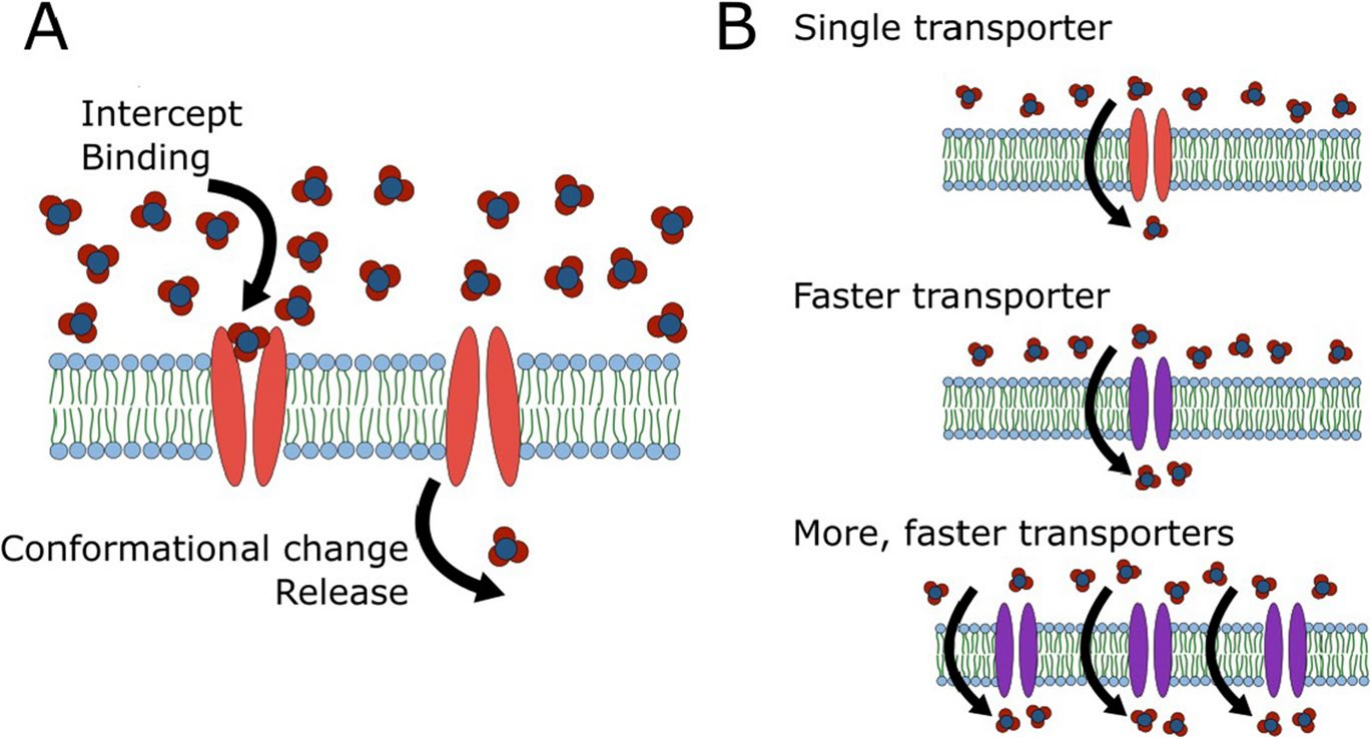
Considerations for how nitrate transporters influence total uptake rate. At the transporter property level (A), significant knowledge gaps remain about how the handling time is influenced by a specific transporter's ability to intercept and bind nitrate ions, the speed of conformational change, the ability to release the ions to the cytoplasm, and to reset to be ready for the next uptake event. At the cellular level (B), knowledge gaps remain about how allelic differences in transporter structure and properties affect uptake (as shown in A), and how different numbers and types of transporters in the cell membrane scale nutrient uptake

Variation in uptake kinetics, maximum velocities, and affinities have been measured across species, genotypes, and even among root classes (York et al. 2016a, b). Together, these imply that various configurations of the molecular machinery or different types of machinery, lead to differences in measurable uptake parameters (Fig. 13). However, what is missing is how this is achieved. Little is known about scaling from a single transporter to the entire root system. Most modeling of uptake in plant root system has utilized Michaelis–Menten kinetics with little consideration of deeper mechanistic questions, such as how the maximum uptake rate is influenced by the number of transporters per unit membrane surface area or the abundance of ATPase. Griffiths and York (2020) proposed a more explicit treatment of transporters in models similar to what has been used in algae that includes number of transporters and their individual handling times (analogous to uptake rates). Major research gaps include quantifying the number of transporters on the root epidermis so that uptake rates can be related to both the number and the individual properties of various transporters.

While deeper mechanistic understanding of nutrient uptake and how it varies would be useful, direct selection on uptake kinetics itself may be possible. For example, Griffiths et al. (2021) developed a moderate-throughput phenotyping platform for quantifying nutrient uptake in nutrient solution. This *RhizoFlux* system was used to screen 26 parents of a maize diversity panel, and found substantial genetic variation for nitrate uptake rates on a *per length* basis. The heritability implies that nitrate uptake rate could be selected directly in a breeding program. This decoupling of uptake rate from total root system uptake could be important to not confound selection with root system architectural parameters. At the same time, Griffiths et al. (2021) also identified a positive relationship of uptake rates with root respiration, indicating the two properties need to be co-optimized as discussed below (Sect. "Phene Integration and Multi-objective Optimization for Breeding Strategies").

Evidence for the functional utility of nitrate transporter kinetics comes from field, lab, and simulation studies. Research extending back to the 1960s characterized nutrient uptake kinetics in various taxa, often within the context of potential application to crop breeding (Griffiths and York 2020). York et al. (2016a, b) demonstrated that variation among root classes in maize for uptake kinetics with increased I_max_ driving increased nitrogen accumulation and shoot mass in silico*.* A 2.3-fold variation in maximum uptake rate (I_max_) was found in maize inbred lines (Pace and McClure 1986). Direct evidence for the utility of increased nitrate uptake kinetics comes from allelic variation in rice that was linked to grain yield (Hu et al. 2015). Future work to discover natural allelic variation for nitrate transporters and breeding for nitrate uptake kinetics is warranted.

## Root plasticity

**Fig. 14:**
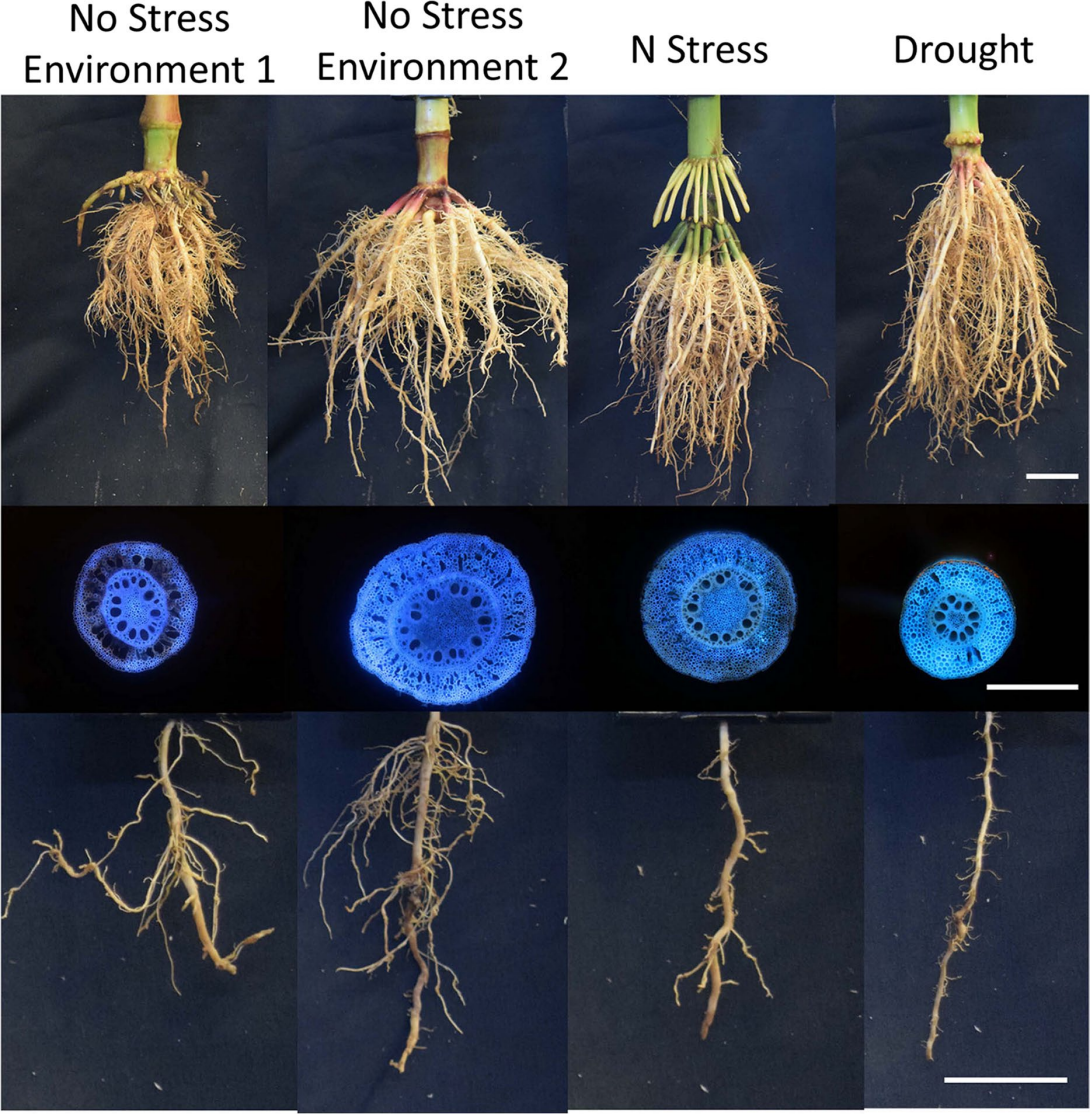
Genotypes vary in their plastic response to environment, nitrogen stress, and drought. Architectural and anatomical images are presented from a single genotype in response to different environments and edaphic stress conditions. Phenotypic plasticity is shown for root architecture, root anatomy, and lateral branching length and density. Scale bar represents 2 cm (root crown and lateral branch) and 1 mm (anatomy). Reprinted with permission from Schneider and Lynch 2020

Generally, plants are plastic (*i.e.* they alter their phenotype) in response to nitrogen availability, and several phenes respond to spatial or temporal changes in soil nitrogen availability (Fig. 14). Plasticity in response to suboptimal nitrogen availability has been observed for a number of root anatomical and architectural phenes. For example, lateral root proliferation in response to nitrate-rich patches is a classic example of nutrient-induced plastic responses of roots (Drew et al. 1975). Maize hybrids form more RCA and have larger cortical cells (Jia et al. 2022), and maize inbreds form more RCA in primary, seminal, and crown roots (Saengwilai et al. 2014a) in low nitrogen conditions when compared to high nitrogen conditions. Some maize inbred and hybrid genotypes respond to low nitrogen by reducing their metaxylem vessel, root cross-sectional, and stele area (Yang et al. 2019) and develop fewer, longer nodal roots with longer lateral branches (Gaudin et al. 2011; Guo et al. 2019). In maize, root growth angles become steeper in low nitrogen conditions (Trachsel et al. 2013) resulting in greater rooting depth. In barley low nitrogen accelerated RCS formation in the field (Schneider et al. 2017a, b). Several studies have also suggested that the form of available nitrogen may influence root plasticity, including lateral root branching densities (Robinson et al. 1988; Meier et al. 2020). However, this may be dependent on the species, phene, and/or the environment (Tran et al. 2014).

Plasticity may be of variable duration in response to an environmental cue (Schneider 2022). Short-term (or physiological) plasticity may allow plants to adjust to temporally variable aspects of the environment. For example, the expression of nitrate transporters fluctuates as a response to nitrogen availability (Feng et al. 2011a, b). In contrast, plastic responses can also involve morphological changes that can be of longer duration. For example, root growth angle is established relatively early in plant development and a plastic response to root growth angle may be beneficial in conditions of sustained edaphic stress (e.g., low phosphorus availability; Zhu et al. 2005) but may be maladaptive in stresses that fluctuate in shorter time scales (e.g., low nitrogen availability) by creating permanent responses to ephemeral conditions. For example, early in the growing season following fertilizer application, the topsoil may have optimal nitrogen availability and thus plants may respond by developing shallow root angles early in plant growth. However, as the growing season progresses, nitrogen is taken up by the plant and leached into deeper soil domains resulting in greater nitrogen availability at depth. Only emerging roots can alter their root angle to respond to changes in soil nitrogen availability, while established roots with a shallow growth angle cannot alter their angle, unless the trajectory of root angle with time changes, possibly limiting their utility for deep nitrogen capture.

Phenotypic plasticity to low nitrogen availability also encompasses both active and passive responses. Active plasticity is generally anticipatory and occurs in response to an environmental cue that involves modification in developmental pathways or regulatory genes (Forsman 2015). For example, steeper root angles or fewer nodal roots may be an active plastic response to low nitrogen availability. In contrast, passive plasticity (also referred to as apparent plasticity) may result from resource limitations, allometry, or ontogeny and generally is not anticipatory or adaptive but a mere consequence of the environment (Weiner 2004; Forsman 2015). For example, in environments with limited nitrogen availability, generally above-ground and below-ground biomass and the quality of reproductive organs are reduced. However, this reduced growth in response to the environment is typically considered a type of passive plasticity since it is a consequence of inevitable resource limitations and physical growth conditions.

In addition, many taxa change their biomass allocation during ontogeny, and often phenotypes have strong allometric associations. Therefore, environmental factors that influence growth rates or development, such as low nitrogen, may also influence biomass partitioning and allometry. For example, changes in root-to-shoot ratios are associated with nitrogen limitation (Poorter and Nagel 2000), and changes in root-to-shoot ratios are often explained by the functional equilibrium theory, or prioritizing and optimizing the acquisition of resources in a manner that maximizes plant growth (Poorter and Nagel 2000). For example, root-to-shoot ratios generally increase in response to low nitrogen availability, and this may be considered an adaptive response as root growth is prioritized to obtain the most limiting resource. However, younger plants generally have a greater root-to-shoot ratio, and ontogeny may explain this plastic response if nitrogen limited plants lag behind developmentally. Therefore it is difficult to interpret plasticity such as changes in root-to-shoot ratios, as they may reflect smaller plant size and, therefore, passive plasticity and not an adaptive response (Correa et al. 2019).

In many cases, the adaptive value of phenotypic plasticity can be difficult to interpret and is environmentally dependent. In the field, plants are often exposed to stress factors that are spatially and temporally dynamic. For example, in typical high-input agroecosystems, seeds are planted in the topsoil which is rich in nitrogen from fertilizer application. However, throughout the growth season, nitrogen may leach into deeper soil domains resulting in relatively greater nitrogen availability in deeper soil domains. The investment of carbon and nutrient resources in root tissue construction and maintenance early in plant development may limit the opportunity for the construction of additional root length in deeper soil domains as resource availability changes. If roots proliferate early in the growth season in fertilizer-rich topsoil, this limits the opportunity for construction of roots in deeper soil domains later in the growth season where nitrogen resources are likely to be located (Schneider and Lynch 2020). However, nitrogen capture early in plant development would drive shoot growth and thereby increase photosynthate availability for root growth, which could increase rooting depth over time. This case illustrates the complexity of the fitness landscape of root plasticity.

However, many root plastic responses are adaptive in response to low nitrogen availability, including the increased formation of RCA or the development of fewer nodal roots (Saengwilai et al. 2014a; Guo et al. 2019). It has been proposed that adaptive plasticity is the future of crop breeding since it would enable the development of more efficient crops that could adapt to changing environments (Lobet et al. 2019). Adaptive plasticity may promote the establishment and persistence of crops in novel environments and allow genotypes to have broader tolerance and greater fitness across a range of environments (Schneider 2022). Understanding the genetic and mechanistic basis of phenotypic plasticity may enable the rapid development of more productive crops for future environments (Topp 2016). The adaptation of genotypes to sudden environmental changes, like those caused by human disturbance or policy changes (e.g., fertilizer regulations), could also be an advantage of plasticity since these changes generally occur at too rapid of a pace for an evolutionary response or the development of new cultivars through breeding. However, organisms may not be able to express plasticity that is entirely adaptive in response to nitrogen stress, indicating that there are tradeoffs, costs, or limits to the expression of plasticity. Many of these tradeoffs and costs have been the subject of speculation (DeWitt 1998; Relyea 2002; Schneider and Lynch 2020). However, the mechanisms and evidence for this are lacking in the literature.

It has been speculated that phenotypic plasticity was a useful mechanism for crop ancestors and landraces to grow and develop in environments that were unmanaged, unfertilized, and non-irrigated. In these natural ecosystems, plasticity may be advantageous by enabling the plant to exploit resource patches, for example, by increasing lateral root proliferation. However, in modern, high-input agroecosystems, plasticity may come at a greater cost than benefit as many constraints to plant growth and soil resource acquisition have been mitigated through use of fertilization, irrigation, and pesticides. In high-input agroecosystems, parsimonious, non-plastic root phenotypes including fewer axial roots, reduced density and length of lateral roots, reduced cortical cell file number and cell size, and reduced cortical parenchyma through aerenchyma formation and senescence may be beneficial for nitrogen capture by permitting deeper rooting (Lynch 2018). Root phenotypes that explore deep soil domains, whether through plastic responses or not, may enhance the capture of deep resources like nitrogen in most agroecosystems (Manschadi et al. 2006; Henry et al. 2011).

Root anatomical, architectural, and physiological phenes express a wide range of plastic responses to soil nitrogen availability. These plastic responses vary in duration and adaptive value, and their influence on plant fitness depends on interaction with other root phenes and the environment. Phenotypic selection for plasticity may be a viable strategy in breeding programs, however selection should occur under specific target environments or edaphic stresses, as small environmental changes may significantly influence the utility of plasticity. In addition, phenotyping for plasticity should be evaluated for individual phenes rather than phene aggregates, as the expression of individual phenes also influences the utility of plasticity. The selection of genotypes that are plastic to a wide range of environments and stresses may be maladaptive in environments with multiple, dynamic stresses. The fitness landscape of plasticity is highly complex, yet poorly understood and merits further research to understand the utility of plasticity for nitrogen capture in a range of environments.

## Harnessing the rhizosphere microbiome for improved nitrogen capture

**Fig. 15:**
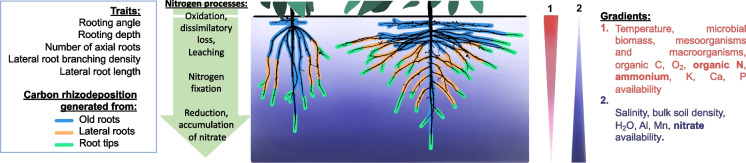
Hypothesized modifications of microhabitats in the endosphere and rhizosphere caused by vertical soil gradients, shown as narrow triangles (on right) to indicate the direction of the gradient. The presented gradients represent available pools of some resources in the most representative agricultural soils globally, although changes in direction or intensity might be observed depending on agricultural management and soil properties. Two common-bean root systems with contrasting root architectures (modified from two actual root images courtesy of Dr. Magalhaes A. Miguel). The colors around the roots represent different origins of carbon rhizodeposition, with old roots shedding tissue from secondary growth and root exudates. Deep rooting systems deposit different composition of exudates in deeper soil domains due to the increase in root tips in deeper locations. Nitrogen cycling predominant processes and their changes along the soil profile are depicted on the big green arrow. Details on the specific root phenotypes and their interactions with microbes are provided in the text. Modified from Galindo-Castañeda et al. 2022

The rhizosphere is the soil actively influenced by root activity (definitions of rhizosphere and rhizosphere processes are available in Hinsinger et al. (2009), York et al. (2016a, b), Schnepf et al. (2022)). This interface between roots and soils is the subject of a growing amount of research on soil nitrogen cycling and uptake in crops (Moreau et al. 2019). Root-microbe associations in the rhizosphere could play an important role in plant nitrogen uptake given that microbes harness a wide range of enzymes that catalyze the transformation of nitrogen-containing compounds in soils (Kuypers et al. 2018) and because microbes participate in the regulation and activity of nitrogen transport from the rhizosphere to the root cortex (Zhang et al. 2019; Hui et al. 2022). Recent research has demonstrated that root architecture (Yu et al. 2021) and anatomy (Salas-González et al. 2021, Galindo-Castañeda 2018, Galindo-Castañeda et al. 2023) interact with rhizosphere microbes under low nutrient supply, but the mechanisms and relevance of the activity of nitrogen cycling microbes and their associations with adaptive root anatomical and architectural phenotypes in crops is poorly understood. Synergies and tradeoffs of microbial associations as influenced by root anatomy and architecture to optimize nitrogen capture by crops in agroecosystems are promising avenues for crop breeding and microbiome engineering (Galindo-Castañeda et al. 2022). Usually, the adaptive value of root phenotypes is considered separately from microbial processes, but this view has started to change in recent years with the demonstration that feedbacks exist between roots and microbes (Salas-González et al. 2021), which we propose may lead to benefits or tradeoffs when selecting for specific root phenotypes. In this section, we discuss microbial rhizosphere processes that interact with root adaptations relevant for nitrogen capture as described in other sections of this perspective. We consider possible effects of these root adaptations on nitrogen-cycling processes performed by microbes such as N_2_ fixation, nitrification, denitrification and ammonia oxidation (Kuypers et al. 2018), which could be relevant for plant nitrogen uptake. We argue that indirect and direct selection of such root phenotypes through plant breeding would have associated synergies or tradeoffs for microbial associations. We compile our hypotheses and research gaps in Tables 2 and 3 and we show the gradients and hypothesized nitrogen processes in soil profiles in Fig. 15.

**Table 2:** Root anatomical phenes with their states and the hypothesized effect on conditions from microbial habitats and the expected effect on nitrogen-microbial transformation

Root phene	Phene states	Hypothesized effect on root exudation, physicochemical conditions, and habitat space for microbes	Hypothesized effect on nitrogen dynamics in the rhizosphere	Supporting evidence
Root diameter	Thicker vs Thinner	• Increase in surface area per root length for microbe attachment and root exudation • Reduced total amounts of exudates per root mass or length unit • Reduced and/or different composition of exudates • Increase amount of shedding tissue per root length unit	• May reduce the rate of nitrogen uptake by root length unit and reduce the surface area available to associate nitrogen-cycling microbes	(Galindo-Castañeda et al. 2019; Saleem et al. 2016; Wang et al. 2017; Zai et al. 2021)
Cortical cell size	Large vs Small	• Reduced apoplastic and symplastic pathway length for exudates to be released into the rhizosphere • Increase AM colonization	• Increase probability to obtain more nitrate if AM-regulated nitrate transporters are upregulated	(Galindo-Castañeda et al. 2019; Hui et al. 2022)
Cortical aerenchyma or cortical senescence	Augmented vs Reduced	• Reduced apoplastic and symplastic transport of exudates into the rhizosphere • Increase of N_2_ and O_2_ concentration in the rhizosphere	• Favor aerobic metabolism therefore nitrification, ammonia oxidation, ammonification	(Arth and Frenzel 2000; Galindo-Castañeda 2018, 2019; Li et al. 2008; Risgaard-Petersen and Jensen 1997)
Apoplastic barriers and lignified layers in the hypodermis	Present vs Absent	• Reduced amounts of exudates transported to the rhizosphere • Barriers for microbial colonization in the cortex or selection towards organisms with the capability to degrade these barriers (likely pathogens?)	• Reduced exudation in the rhizosphere, which changes the nitrogen cycling predominant reactions towards a lower N2:NH4 ratio with low C availability to e- acceptor	(Fröschel et al. 2021; Salas-González et al. 2021; Ishii et al. 2011)
Root hair density and length	Long, dense vs Short, scarce	• Greater amounts of exudates in the rhizosphere • Extended surface for microbial attachment • Steeper gradients in nutrients in the rhizosphere due to increase nutrient uptake by root hairs, favoring microbial diversification	• Increase production of nitrification inhibitors in plants that produce these compounds • Increase nitrogen availability in general for the plant, which will indirectly benefit exudation in the rhizosphere	(Brown et al. 2013; Burak et al. 2021; Dayan et al. 2009; Gebauer et al. 2021; Holz et al. 2018; Robertson-Albertyn et al. 2017; Saengwilai et al. 2021; Schweiger et al. 1995)

**Table 3:** Root architectural phenes or traits with their states and the hypothesized effect on conditions from microbial habitats and the expected effect on nitrogen-microbial transformation

Root phene or trait	Phene or trait states	Hypothesized effect on root exudation, physicochemical conditions, and habitat space for microbes	Hypothesized effect on nitrogen dynamics in the rhizosphere	Supporting evidence
Rooting angle and depth	Steep – deep roots vs shallow – shallow roots	• Increase in percentage of fixed carbon allocated to deeper soil layers • Increase of microaerophilic or anaerobic pockets around the rhizosphere of root tips • Soil compaction affecting the rhizosphere	• Increased number of root tips under microaerophilic conditions, favoring reduction of nitrogen compounds to ammonia or to N_2_ • Nitrification may be favored in the rhizosphere of plants containing aerenchyma through which oxygen can come into deeper soil domains • Nitrogen fixation may occur in the rhizosphere where air is brought to deep soil layers. The resulting ammonia might be quickly taken up by the plants rather that entering the dissimilatory N_2_ production	Not reported in literature –Nitrogen cycle in paddy rice as it relates to soil depth: Ishii et al. 2011
Number of axial roots	Many vs few	• Increased amounts of root exudates • Increase oxygen diffusion from the roots that have increase aerenchyma • Increase surface for microbial attachment • Larger amounts of substrate to be degraded from root decay • Steeper gradients of nutrient concentration in the depletion zone in the horizontal axis	• Reduced ammonia availability due to plant uptake might favor competence with microbes • Oxygen brought with the roots (specially those with aerenchyma) might trigger nitrification of C sources such root exudates or other sources such as debris or organic matter	Not reported in literature – plant–microbe competence for nitrogen: (Moreau et al. 2015)
Lateral root density	High vs Low	• Greater number of exudation points from lateral roots • Greater number of attachment points for microbes • Steeper gradients of nutrient concentration in the depletion zone in the horizontal axis	• Diversification in metabolism, including nitrogen cycling reactions, under more pronounced nutrient and water gradients in the depletion zones around the lateral roots • Reduced ammonia availability due to plant uptake might favor competence with microbes	(Schmidt et al. 2018)—plant–microbe competence for nitrogen: (Moreau et al. 2015)
Lateral root length	Long vs Short	• Root exudates allocated farther from the axial root, or from roots of lower-branching orders. Overall increase of exudates per plant	• Longer roots may recruit a more diverse microbiome due to the increased soil volume explored • Attenuated effect of intra-root competition for nitrogen, which may lead to reduced plant–microbe competition for nitrogen. This may lead to increase mineralization of organic sources of nitrogen by the microbes, which are distant from the main axial root	Not reported in literature. Studies on lateral vs axial roots: (Saleem et al. 2016; Zai et al. 2021)

### Rhizosphere microbial-driven nitrogen cycling and root anatomy

Root anatomy is associated with microbial colonization (Garrett 1981; Dreyer et al. 2010; Galindo-Castañeda et al. 2019; Salas-González et al. 2021; Zai et al. 2021; Tables 2 and 3). This interaction may come from the microhabitats determined by root anatomy, by the physicochemical gradients that occur from the bulk soil towards the rhizosphere and root epidermis, and by changes in the molecular interactions between microbes and plant cells in the rhizosphere and the root cortex. Perhaps the most influential phene that may affect nitrogen cycling is RCA because it changes the redox potential of the rhizosphere (reviewed by Hinsinger et al. 2009, Fig. 16), which strongly influences the prevalence of a given reaction within the microbial nitrogen cycle (Kuypers et al. 2018). When plants have more RCA, more oxygen diffuses to the rhizosphere and production of nitrate, nitrite and nitrous oxide would be favored. This was shown partially in rice, with a genotype expressing more RCA having increased nitrification compared to a genotype that had reduced RCA (Li et al. 2008). Rapid plant uptake of the resulting nitrate would result in a benefit, but partial oxidation to nitrite and nitrous oxides would result in the loss of nitrogen from the system. Alterations in microbial colonization of the cortex due to RCA (Galindo-Castañeda et al. 2019) may cause a reduction in nitrogen capture when the symbiosis is contributing to it.

**Fig. 16:**
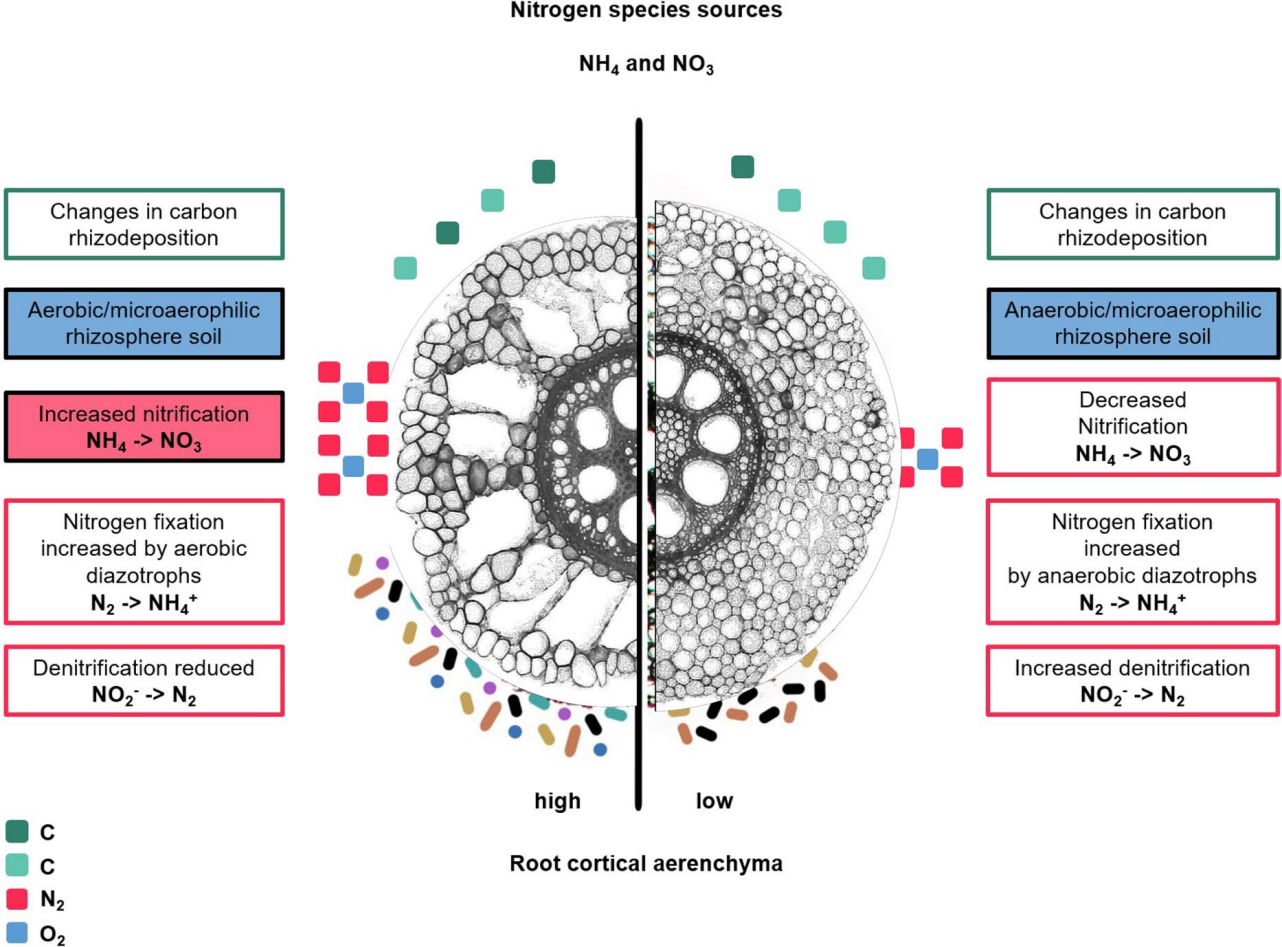
Hypotheses about changes in diversity, abundance and nitrogen transformation capacity of the bacterial community in the rhizosphere of maize due to changes in RCA. The text boxes describe hypotheses about root-derived carbon, air, and nitrogen transformation. Carbon rhizodeposition is represented by green square icons; the number of icons is related to the hypothesized change in amount of C deposited at each RCA phenotype. The more icons, the more carbon. The differences in green tones indicate changes in biochemical composition. Air is represented by blue and pink square icons, corresponding to O_2_ and N_2_, respectively. The number of icons represent the hypothesize change in amount of air. The colored bastons and circle icons represent bacterial communities and their different colors represent diversity (more colors, greater diversity); the number represent the expected microbial abundance (not to scale or proportional to the actual amounts). The hypothesis for which experimental support exists are in color-filled text boxes (Risgaard-Petersen and Jensen 1997; Arth and Frenzel 2000; Kennedy et al. 2004; Li et al. 2008; Galindo-Castañeda 2018), while hypothetical statements are written in non-color-filled text boxes. Taken (unchanged) from Galindo-Castañeda et al. 2022

Other mechanisms of microbe-plant interactions where anatomy could be important for nitrogen cycling is the expression and functioning of nitrogen transporters as well as the regulation of root exudation. How these rhizosphere processes are associated with microbes participating in the nitrogen cycle remains elusive. The relative abundance of ammonia oxidizing Archaeans of the genus *Nitrososphaera* was significantly associated with high RCA expression under low nitrogen conditions in field grown maize (Galindo-Castañeda 2018; Galindo-Castañeda et al 2023). These results suggest that maize with more RCA may favor nitrification, perhaps through the transport of oxygen to the rhizosphere using the RCA channels, similarly to rice (Li et al. 2008). Selection of cereal cultivars with increased RCA may therefore stimulate nitrogen mobilization from organic reservoirs by providing oxygen in the rhizosphere. This could lead to better nitrogen availability if the main source of nitrogen is organic matter.

Apoplastic barriers formed by suberin deposition in the intercellular spaces of the endodermis and hypodermis are common to protect roots from pathogens (Garrett 1981; Schreiber et al. 1999), desiccation, and oxygen loss (Song et al. 2023). Suberin formation in the endodermis has been demonstrated to be coordinated by feedbacks between roots and microbes in *Arabidopsis thaliana* grown in low phosphorus (Salas-González et al. 2021). However, it is not known if similar mechanisms exist under low nitrogen availability in *Arabidopsis*, other model plants or in crops although there are indications that root nitrate uptake is modified by suberin formation (Plett et al. 2016; Melino et al. 2021), and that exudation changes with suberin production (Durr et al. 2021). We propose that increase in suberin deposition could result in a reduction in carbon deposition in the rhizosphere, which would be linked to a net reduction of microbial activity in the rhizosphere.

Root hairs change the area, location, and type of attachment surface of microbes to the roots and have effects on root microbial communities (Tables 2 and 3). Some direct effects of root hairs on rhizosphere ecology that would be relevant for nitrogen capture are increased production of root exudates with longer and denser root hairs (Holz et al. 2018), and the increase of the complexity and extent of the rhizosphere volume (Burak et al. 2021). Root hairs have been proposed as a significant root phenotype determining the biodiversity and abundance of rhizosphere bacterial communities in cereals (Robertson-Albertyn et al. 2017; Gebauer et al. 2021). Increased length and density of root hairs could have synergistic effects with microbes participating in nitrification and ammonification of organic matter by offering extended surface for these microbes to attach to the root, while increasing soil exploration. However, having more root hairs may lead to an increase in carbon allocation to the roots through exudation (Holz et al. 2018), which would increase root metabolic costs. Although analysis of wild-type and hairless mutants of *Arabidopsis* under conditions of suboptimal phosphorus availability showed that mutants lacking root hairs had reduced root respiration (Bates and Lynch 2000a), this effect may have been confounded by reduced phosphorus uptake in hairless mutants, and any additional metabolic costs of root hair formation are far outweighed by benefits for plant phosphorus capture, growth, and competitiveness (Bates and Lynch 2000a, b, 2001). It was recently shown that maize genotypes with longer root hairs have substantially better nitrogen capture (Saengwilai et al. 2021), which again indicates that any additional metabolic cost of producing root hairs is outweighed by greater resource capture.

### Rhizosphere microbes participating in the nitrogen cycle and root system architecture

Root system architecture determine the niches for nitrogen-cycling microbes in the rhizosphere. Vertical soil gradients regulate oxygen concentration, availability of nitrogen and other nutrients, water availability, and temperature (Fig. 15). Therefore, rooting depth and the architectural and anatomical phenotypes that affect it could influence the metabolism of nitrogen cycling microbes. Shallow root systems may offer a better habitat for nitrogen acquisition from organic matter in the topsoil. Hypothetically, intermediate and deeper root systems could offer a better habitat for nitrification given leaching of nitrate to deeper soil domains, and for nitrogen fixation given the deleterious effect that oxygen has on the nitrogenase enzyme. In addition, the reduced redox potential of deeper soil domains in comparison with shallower soil would favor nitrate reduction if oxygen is not supplied through aerenchyma, or by means of biopores.

Lateral root density and length are probably the two most important architectural phenotypes regulating root microbial associations given that lateral roots comprise the large majority of root systems, and the effect that they have on exudation zones, attachment surface, and the location along both vertical and horizontal gradients of carbon exudation. Roots with increased lateral root branching density would have greater carbon deposition which could boost nitrification and ammonification of such exuded compounds and may prime the rhizosphere soil (or the soon-to-be) rhizosphere soil to start these processes. Steeper nitrogen gradients resulting from greater root density per unit soil volume, due to high lateral root branching density, may also affect the environment for nitrogen cycling prokaryotes by stimulating organic matter degradation either anaerobically or aerobically.

Another aspect of this phenomenon is that several plant growth promoting microorganisms cause changes in root architecture (e.g. Contreras-Cornejo et al. 2009; Garnica-Vergara et al. 2016; Patten and Glick 2002; Bashan and de-Bashan 2010; Torres et al. 2018; Zúñiga et al. 2013). These interactions are complex and involve the production of phytohormones by the microbial partner, or modification in phytohormone perception by the plant (Verbon and Liberman 2016; Frankenberger and Arshad 2020). Although there is considerable research on the potential and basic mechanisms of phytohormone-mediated plant microbe interactions, the development of agricultural technologies based on such interactions remains limited (Hungria et al. 2022; Wen et al. 2021, Raymond et al. 2021). For example, how and when plant genetic determinants and plasticity in the production of new lateral roots interact with the phytohormones produced by microbes to control lateral root branching is poorly understood. Possible cues to root architectural plasticity could possibly originate from newly mineralized nitrogen, or just metabolized nitrogen-containing compounds by microbes. A single-sided perspective where plants are inoculated with microbes to force the plant to produce more lateral roots would have tradeoffs in terms of the energy cost to the plant in maintaining such associations under abiotic stress, since reduced lateral root density is associated with greater adaption to water deficit and low nitrogen bioavailability (Sect. "Axial root number"). It is reasonable to wonder if failures in obtaining yield increases after inoculation with specific plant-growth promoting microorganisms are related to imbalances in the resource economy of plants under limiting nitrogen conditions. For example, if microbial inoculants increase lateral root branching density, the high metabolic cost of this effect to the plant should be considered as a possible tradeoff of inoculation. Studies determining the risks, metabolic costs, and opportunity costs of inoculation with plant-growth promoting microorganisms are lacking, yet urgently needed.

### Interaction of microbes with nitrogen transporters in the root cortex

Nitrate and ammonium transporters in the root epidermis influence the rate of nitrogen uptake (see Sect. "Nitrogen Uptake Kinetics: Scaling From Transporters to the Root System"). The expression and functioning of these transporters are sometimes modified by root microbial colonization, as in the case of arbuscular mycorrhizal (AM) fungi and ammonium transporters in maize (Hui et al. 2022), or the rhizosphere microbiome and a nitrate transporter in rice (Zhang et al. 2019). This implies that root anatomical phenotypes associated with arbuscular mycorrhizal colonization may play a role in the capability of roots to transport ammonium or nitrate. Cortical cell size and the size of aerenchyma lacunae were directly correlated with AM colonization in maize hybrids growing under high nitrogen fertilization (Galindo-Castañeda et al. 2019). Although larger cells and reduced RCA may promote AM colonization, and thereby the expression of nitrogen transporters on the root surface, reduced RCA increases root metabolic costs, which is detrimental for resource capture, although RCA forms in older root tissue that is generally less active in resource capture (Sect. "Anatomical Phenotypes that Reduce the Metabolic Cost of Soil Exploration Improve Nitrogen Capture"), which illustrates the complexity of the fitness landscape for root phenotypes.

If microbes have the potential to modify the expression of nitrate or ammonia transporters on the root surface, the next question is where this is occurring and what anatomical or architectural phenotypes favor such associations. Anatomical phenotypes such as reduced epidermal suberization, increased root hairs, and increased root diameter could have synergistic effects with microbial-triggered expression of nitrogen transporters by reducing physical barriers and increasing the surface where the transporters are to be expressed. Architectural phenotypes such as increased lateral root branching density could also favor increased expression of transporters. Ultimately, root growth angle and associated rooting depth distribution would dictate the utility of such transporters by locating them in soil domains with available nitrogen.

### Nitrogen fixation and root phenotypes

Biological nitrogen fixation (BNF) is the conversion of atmospheric N_2_ into ammonia by the enzyme nitrogenase, present only in prokaryotes (Burris and Roberts 1993). Synergies or tradeoffs of selecting root architectural or anatomical phenotypes for symbiotic or associative BNF in crops are understudied. Symbiotic BNF in higher plants occurs in root nodules formed when diazotrophic bacteria interact with the host root. The anatomy of the formation and development of root nodules and the regulatory mechanisms of the symbiosis, especially in legumes, have been well characterized (de Bruijn 2015; de Bruijn and Hungria 2022). However, the relation between nodulation and natural variation of root architecture and root anatomy within individual plant populations has been rarely addressed. It is important to discover synergies or tradeoffs of possible adaptive root phenotypes for nitrogen uptake and nodulation to select optimal combinations of rhizobia and root phenotypes. For example, a synergistic interaction was identified between shallow root angle and rhizobia BNF in an inbred population of soybean, with plants showing shallow angles positively correlated with nodule formation and nitrogen content in field-grown soybean (Yang et al. 2017). These results suggest a possible tradeoff of targeting deep-rooting systems in soybean breeding programs. If deep-rooting systems are to be selected in legume crops, a concomitant study on nodulation and contribution of BNF is recommended. Possibly, conditions present in deep soil domains are not suitable for the symbiosis or BNF by rhizobia and therefore a substantial portion of nitrogen that could be available through BNF may be lost for deep-rooting plants. Root architectural and anatomical phenotypes, including those covered in the present perspective, and their interactions with BNF should be explored.

A more challenging research question is if associative BNF also interacts with root system architecture, and moreover with root anatomy. The visualization and measurement of associative BNF in the rhizosphere is more involved and less well characterized than symbiotic N_2_ fixation due to the lack of nodules in associative BNF. The problem becomes more complicated with the fact that several of the well-characterized free-living N_2_ fixing prokaryotes have other mechanisms to promote plant growth (see Sect. "Rhizosphere Microbes Participating in the Nitrogen Cycle and Root System Architecture") that cause, among other things, changes in root architecture. It is therefore difficult to differentiate the direct effect of BNF on fixing N_2_ from the indirect effect of modifying root architecture to improve nitrogen uptake. Nevertheless, the contribution of associative BNF has been estimated or certain bacterial strains (Santi et al. 2013) but little has been reported on the impact of N_2_ fixation on the total nitrogen budget in crops, with the exceptional case of *Azospirilum brasilense* inoculation in several cereals in Brazil (Hungria et al. 2022). Benefits of inoculation in maize with strains of this species indicate that up to 25% of nitrogen fertilization could be replaced by inoculation. The mechanism of plant growth promotion in this case was mostly attributed to the increase in root volume and branching without an increase in root biomass, rather than to the increase in available N_2_ in the rhizosphere, but the amount of fixed N_2_ was not measured. Diazotrophs may be more abundant under microaerophilic conditions favored by root architectures intermediate between shallow and deep, or in rhizospheres with reduced oxygen diffusion but with enough air supply of air to obtain N_2_.

### Biological nitrification inhibition

Nitrification, the conversion of ammonia to nitrate, is a natural microbial process that occurs in agricultural soils at high rates, causing nitrogen losses in agroecosystems (Ladha et al. 2005). The production of nitrification inhibitors has been considered as a possible target for breeding plants with better nitrogen efficiency (Subbarao et al. 2009a; Canfield et al. 2010). Crop plants such as wheat, rice, and sorghum produce biological nitrification inhibitors, which differ in chemical composition and modes of activities, providing diversity in biological nitrification inhibitors in agricultural soils (reviewed in Coskun et al. (2017a, b)). Little is known about the location of exudation of biological nitrification inhibitors within root systems, although they are produced only when ammonium is present in the growth media (Subbarao et al. 2009b). This leads to the hypothesis that exudation of biological nitrification inhibitors could occur mostly in the topsoil, where ammonium is more abundant and newly generated from organic matter degradation. Exudation of biological nitrification inhibitors in sorghum occurs through root hairs (Dayan et al. 2009), which is an indication also of the importance of root anatomy in the process of biological nitrification inhibition. Many questions remain regarding the interactions of biological nitrification inhibitors with root architecture and anatomy because most studies have been performed in hydroponics and the research questions do not consider root anatomy or architecture. Increased root hair length and density is an important phenotype to investigate interactions of root anatomy with nitrification inhibitors, although rooting depth would also be logical to investigate given the gradient in concentrations of nitrogen compounds by soil depth. Further, plant responses to the increased ammonium resulting from the nitrification inhibition such as the proliferation of lateral roots (Wu et al. 2022) would be interesting to assess in the cereals producing these compounds. This is a whole field of research open to be explored, with implications for targeting adaptive phenotypes in crop breeding that will reduce nitrogen losses in agricultural fields.

### Nitrogen acquisition mediated by arbuscular mycorrhizal symbioses

The arbuscular mycorrhizal symbiosis is common in crop plants and is well known to benefit the acquisition of diffusion-limited nutrients, especially phosphorus, by extended the effective diffusion depletion zone in the rhizosphere (Smith and Read 2008). Naturally, soil fungi including arbuscular mycorrhizal fungi are capable of acquiring nitrogen from the soil, and transporting nitrogenous compounds across their membranes. It is therefore unsurprising that plant symbionts can acquire nitrogen via their fungal symbionts in the arbuscular mycorrhizal symbiosis (Tanaka and Yano 2005; Govindarajulu et al. 2005; Jansa et al. 2019a, b; Dierks et al. 2022). The importance of this pathway in the field is unclear. Several root anatomical phenotypes may affect the arbuscular mycorrhizal symbiosis by altering the extent or persistence of root cortical tissue, including root cortical senescence (Schneider et al. 2017a, b), root cortical aerenchyma (Galindo-Castañeda et al. 2019, 2022), and root secondary growth (Strock et al. 2018). If this pathway is important for nitrogen capture in the field, it is possible that variation for these phenotypes may improve nitrogen capture, although suboptimal phosphorus availability is common globally, so any benefit from improved nitrogen capture may be conflated with improved root growth and soil exploration resulting from alleviation of phosphorus limitation.

## Integrated root phenotypes for improved nitrogen capture

The utility of root phene states for nitrogen capture is a function of their direct effects as well as their interaction with other phene states in integrated phenotypes, and in turn how the integrated phenotype interacts with its environment (York et al. 2013; Lynch 2022b). Phene interactions may be synergistic, *i.e.* resulting in greater than additive effects on nitrogen capture, neutral, *i.e.* resulting in simply additive effects on nitrogen capture, or negative, *i.e.* resulting in less than additive effects on nitrogen capture. Phene interactions have not received much research attention, due in part to the large number of potential combinations of underlying phene states in integrated phenotypes, and the difficulty of empirically generating plant phenotypes that possess specific phenotypic combinations. However, several empirical and in silico studies indicate that interactions among root phenes are important determinants of the capture of soil resources, including nitrogen.

**Fig. 17:**
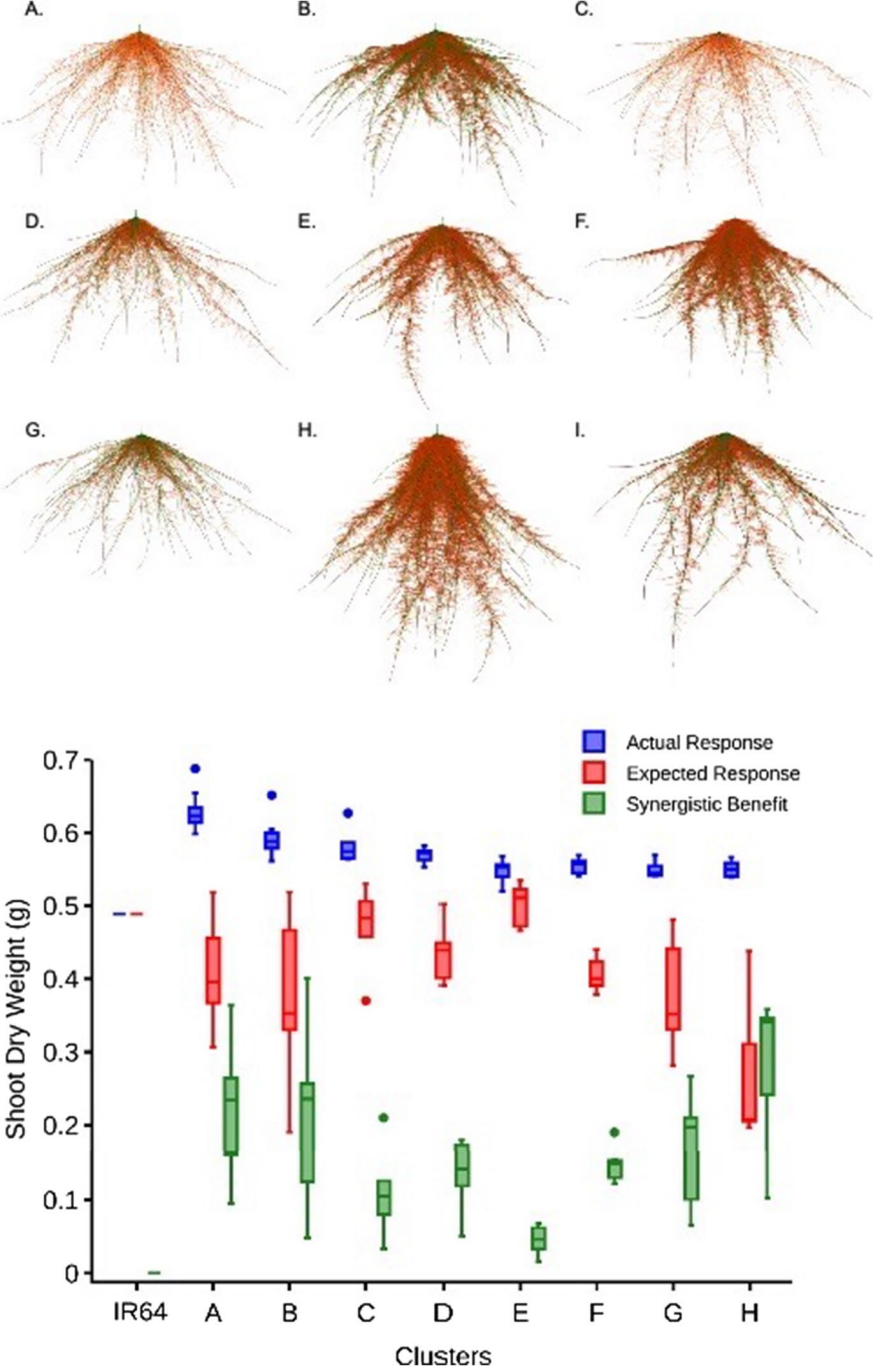
Top panel: *OpenSimRoot* visualizations of representative rice root phenotypes corresponding to eight architectural clusters (A-H) with the reference phenotype IR64 (I) at 30 days after germination. Bottom panel: Shoot dry weight at 30 days after germination of these phenotypes under nitrogen limitation, showing actual response (simulated biomass), expected response from the additive effects of each phene state in the integrated phenotypes, and synergistic benefits, i.e. growth responses beyond those expected from additive effects. From Ajmera et al. 2022

A study with *OpenSimRoot* identified several rice root phenotypes with superior nitrogen capture in low nitrogen soils in present and future climates (Fig. 17) (Ajmera et al. 2022). Interestingly, the superiority of these phenotypes in comparison with the reference rice phenotype IR64 was due entirely to phene synergisms rather than additive effects (Fig. 17). Multiobjective genetic optimization with *SimRoot* identified optimal root phenotypes of maize and bean for low nitrogen soils, which showed interacting effects of root growth angles, root number, and lateral branching density to create optimal integrated phenotypes (Rangarajan et al. 2022). In silico analysis showed that the benefits of RCA formation for nitrogen capture depended upon lateral root branching density (Postma and Lynch 2011a). Similarly, RCS had greater utility for nitrogen capture in silico in phenotypes with fewer tillers and fewer lateral branches due to decreased intra-root and inter-root competition (Schneider et al. 2017a). Field studies indicate that several root architectural and anatomical phenes interact to improve nitrogen capture, and that maize breeding over the past century has inadvertently selected for integrated root phenotypes with superior nitrogen capture in modern production environments (York and Lynch 2015; York et al. 2015). Integrated phenotypes for improved nitrogen capture also include shoot phenotypes (York et al. 2022). A common pattern among these reports is that phene states that regulate the metabolic costs of soil exploration, such as the number of root axes produced or anatomical phenotypes that reduce the metabolic cost of single root segment, interact with each other since they draw upon the same pool of limited plant resources (York et al. 2013). Another cross-cutting concept is that phenes that position root foraging in soil domains with the greatest resource availability will interact with phenes that regulate the exploitation of those domains, as is the case for root hairs and root growth angle for phosphorus capture in common bean (Miguel et al. 2015). Although phene interactions are poorly understood, they are clearly important for nitrogen capture and merit greater attention.

The large array of root phene states interacting with each other and with the environment results in a vast and complex fitness landscape. The dimensionality of the resulting phenome space far exceeds the capacity of empirical research, especially considering that many of the phenotypes of interest may not exist in nature, nor do many environments of interest, including future climate scenarios. In this context in silico approaches are needed (Sect. "Exploring the Fitness Landscape of the Root Phenome in silico").

## Low-input vs. high-input systems

A number of environmental and management factors influence nitrogen regimes and consequently the fitness landscape of root phenotypes for nitrogen capture. These include factors affecting nitrogen leaching regimes such as precipitation, soil temperature, soil texture, and soil structure, as well as factors influencing root growth and competition for nitrogen, including soil mechanical impedance, subsoil acidity, hypoxia, planting density, root loss, soil biota, etc*.*, as noted elsewhere in this essay. On a global scale the most important agroecological factor influencing nitrogen regimes is simply the use of nitrogen fertilizer. In general, global agriculture can be divided into high-input systems which receive chemical fertilizer, soil amendments, mechanical tillage, pesticides, and sometimes irrigation, vs. low-input systems, which receive much less if any of these inputs. High-input systems dominate crop production in wealthy nations and in capitalized sectors of middle-income economies, whereas low-input systems include smallholder agriculture in developing nations as well as plantations, rangelands, forestry, etc., in which use of intensive inputs is possible but not economical. Notably, intensive nitrogen fertilization would offset the energy efficiency of biofuel crops (Ruan et al. 2016).

### Root phenotypes for improved nitrogen capture in low-input systems

Suboptimal nitrogen availability is a primary constraint to crop production in most low-input agroecosystems (Sanchez 2002; Lynch 2007, 2019). Root phenotypes that improve nitrogen capture should therefore have significant benefits for crop growth and yield in such systems (Lynch 2019, 2022b). In low-input systems the main source of bioavailable nitrogen is mineralization of organic matter in the topsoil and possibly organic amendments added to the topsoil, which means that nitrogen is initially a topsoil resource that becomes available gradually over time, and is sensitive to topsoil moisture and temperature regimes. Water deficit can dramatically reduce nitrogen mineralization from the topsoil (Deng et al. 2021). Another important factor in low-input systems is that such systems are characterized by multiple constraints to root growth such as acidity, suboptimal availability of phosphorus, potassium, and other nutrients, water deficit, and biotic stress (Lynch 2022a). Ongoing soil degradation is creating more hostile soil environments in many low-input systems, especially in developing nations (Lynch et al. 2022). Root phenotypes for improved nitrogen capture in such systems should therefore not entail significant fitness tradeoffs for these other constraints and ideally would improve adaptation to multiple constraints. Such multifunctional root phenotypes include long, dense root hairs, which are helpful for the capture of nitrogen as well as phosphorus, potassium and other immobile soil resources, form rhizosheaths that improve the environment surrounding the root tip, may increase penetration of hard soil, and may expand interactions with the rhizosphere microbiome (Sect. "Long, Dense Root Hairs Improve Nitrogen Capture"). Several root anatomical phenotypes that reduce the metabolic costs of soil exploration improve root growth and therefore improve the capture of nitrogen as well as water and phosphorus (Sect. "Anatomical Phenotypes that Reduce the Metabolic Cost of Soil Exploration Improve Nitrogen Capture"). In contrast, several root architectural phenotypes have tradeoffs for topsoil and subsoil resources including lateral root length and density, axial root growth angle, and number of axial roots (Sect. "Root Architectural Phenotypes to Improve Nitrogen Capture"). The importance of architectural tradeoffs between topsoil and subsoil foraging may not be critical for nitrogen capture in low-input systems, since nitrogen availability is more of a topsoil resource in such systems than it is in high-input systems, but it is important for water capture, since water tends to be a deep soil resource in many agroecosystems (Lynch 2018). Parsimonious root phenotypes with reduced numbers of axial and lateral roots are beneficial for nitrogen and water capture (Sect. "Root Architectural Phenotypes to Improve Nitrogen Capture"), but may be sensitive to root loss, which is important because of the intensity of biotic stress in low-input systems (Schäfer et al. 2022b).

### Root phenotypes for improved nitrogen capture in high-input systems

**Fig. 18:**
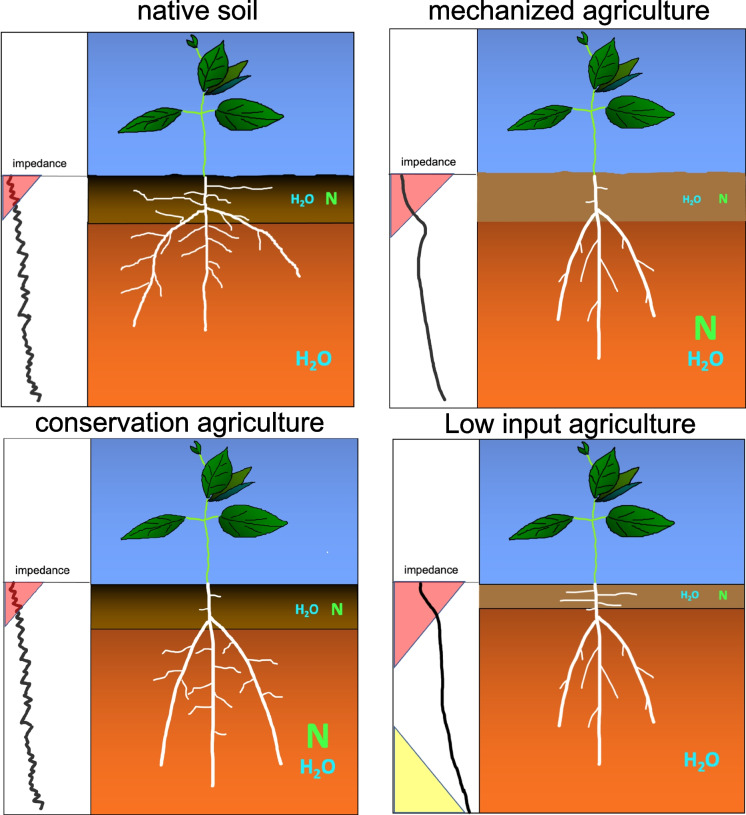
Conceptual scheme of 4 soil scenarios, their impedance profiles, and hypothetical root phenotypes adapted to them, as described in the text. A) Native soil: Mechanical impedance to root growth in native soils is mediated by high organic matter content, low-resistance pathways formed by biopores, soil aggregates, and soil structure, and drought-induced hardening of the topsoil (pink triangle), with nitrogen and water available in the topsoil, but greater water availability at depth. Nitrogen availability is limited and is greater in the epipedon from organic matter mineralization. We propose that root phenotypes adapted to this environment have plastic roots that can respond to local low resistance pathways, and will benefit from dimorphic root phenotypes that promote both topsoil and subsoil foraging. B) Soils under conventional tillage, which in comparison to native soil have a thinner epipedon with less organic matter, hence less water holding capacity and greater susceptibility to soil hardening due to soil drying, fewer low resistance pathways from soil structure and biopores, and a plowpan from vehicle traffic. Nitrogen availability is greater at depth due to nitrate leaching from fertilizer. In these environments, nonplastic root phenotypes that can penetrate through hard surface domains to reach deep soil domains with greater water and nitrogen availability could be advantageous. Root phenotypes that promote topsoil foraging could be less useful for mature plants. C) In high-input agroecologies, traditional tillage in mechanized agriculture is evolving towards reduced tillage in Conservation Agriculture, which will return to some of the features of native soil, including greater topsoil organic matter, greater frequency of biopores, greater aggregate development and improved soil structure, but harder bulk soil, and greater nitrogen availability in deep domains because of nitrate leaching from fertilizer. More plastic root phenotypes that avoid hard, dry soil domains to exploit biopores, soil fissures, and deeper, wetter, and therefore softer soils could be advantageous. Penetrating axial roots, parsimonious root phenotypes, and phenotypes that support subsoil exploration could be useful in exploiting nitrogen and water in deep soil domains. D) Soils under low-input agriculture, with similar characteristics as mechanized agriculture but with greater loss of the epipedon and organic matter, hence greater susceptibility to soil hardening due to soil drying, no plowpan, low nitrogen availability limited to the epipedon because of limited fertilizer use, and the additional barrier of acid subsoil (yellow triangle). In these environments, nonplastic root phenotypes that can penetrate through hard surface domains to reach deep soil domains with greater water availability will be advantageous, along with Al tolerance and dimorphic root phenotypes that also permit capture of shallow nitrogen from mineralization. From Lynch et al. (2022).

Whereas improved nitrogen capture would improve crop production in low-input systems, in high-input systems its main benefit would be for reduced input use and environmental pollution. In these systems, nitrogen fertilizer is generally applied in one or several concentrated applications early in the season when plant size and hence plant nitrogen demand and uptake capacity is limited, resulting in significant risk of nitrogen leaching into deeper soil domains. Several root phenotypes improve rooting depth and thereby improve the capture of deep soil nitrogen resources, as summarized in Sects. "Root Architectural Phenotypes to Improve Nitrogen Capture" and "Root Anatomical Phenotypes to improve nitrogen capture". Since water is a primary limitation to crop growth in many agroecosystems, it has been proposed that in high-input ecosystems that lack topsoil constraints such as suboptimal phosphorus availability, root phenotypes that effectively exploit the subsoil, such as the *steep, cheap, and deep* ideotype, could be useful ideotypes for crop breeding (Lynch 2018). Since many root phenotypes that improve water capture also improve nitrogen capture (Table 1), we propose that such ideotypes would improve nitrogen capture as well. In developed nations traditional mechanical tillage is being replaced by Conservation Agriculture management with reduced tillage (Lynch et al. 2022). This is changing the soil physical regimes confronted by roots. In soils under traditional mechanical tillage plowpans often develop that may restrict both root growth and nitrogen leaching. In such environments, phene states that permit axial roots to penetrate hard soil should improve subsoil foraging and nitrogen capture (Fig. 18)(Strock et al. 2022a). In contrast, soils with reduced tillage lack plowpans and possess better aggregate structure and more biopores (Lynch et al. 2022). Water deficit is forecast to intensify as a result of climate change in many high‐input agroecosystems, which will increase the importance of drought‐induced soil hardening, especially in the topsoil, as a key constraint to root growth. It has been proposed that phenotypic plasticity that permits roots to avoid hard, dry soil domains in order to exploit biopores, soil fissures and deeper, wetter and therefore softer soils would be advantageous in this context (Lynch et al. 2022).

## Exploring the fitness landscape of the root phenome in silico

Recent advances have facilitated data collection at several levels of biological organization which are being integrated to provide a more holistic understanding of plants through mathematical and computational modeling. A computational model provides an explicit formulation of a hypothesis that allows one to simulate, predict, and visualize biological processes. Realistic modeling of plant growth is challenging because it occurs on several scales, with overall fitness being an emergent property of all the processes at the whole-plant or stand scale (Chickarmane et al. 2010) as well as the response of the plant to the environment (Lynch et al. 2022). Several root phenotypes have utility under specific soil and environmental scenarios. However, the utility of a phene state depends on its interaction with other phenes as well as the environment (Sect. "Integrated Root Phenotypes for Improved Nitrogen Capture"). In silico tools capable of mechanistically linking root phenotypes to plant fitness provide a practical way to assess the large number of phene interactions with other phenes and with environmental variables, which would otherwise be impossible to explore empirically (Lynch 2011; Rangarajan et al. 2022).

**Fig. 19:**
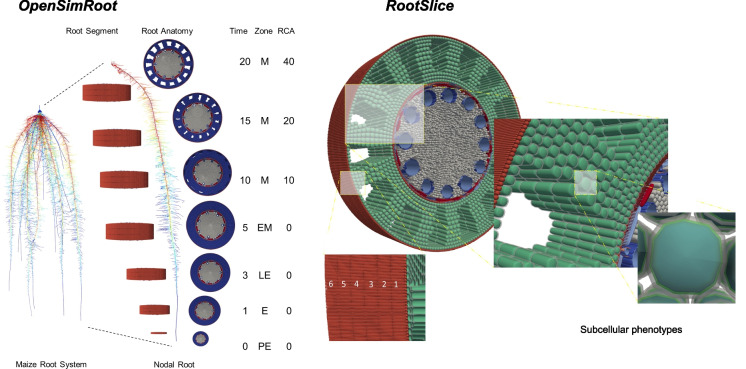
Multiscale modeling from community to subcellular scales exemplified by integration of and *OpenSimRoot/maize RootSlice/maize* models. *OpenSimRoot* visualization of maize root system at 40 days after germination. Color gradient highlights proportion of root cortical aerenchyma, with red to dark blue respectively denoting 40 to 0% aerenchyma formation. *RootSlice* models with different root anatomies corresponding to the five root development zones (i.e., PE: pre-elongation, E: elongation, LE: late elongation, EM: early maturation, and M: maturation) of a maize nodal root were simulated. The matured root undergoes three different levels of cortical aerenchyma formation (i.e., 10, 20, and 40%). In total, seven different root anatomies were simulated in the *RootSlice* model leading to the rhizoeconomic output variables, including root diameter, tissue density, respiration per unit volume, and nitrogen content. Each root zone and corresponding anatomies evolve as the root grows, wherein the cell undergoes the transition from one zone to another. The derived rhizoeconomic variables were temporal scaled with root growth (i.e., transition from one zone to another) and passed to the *OpenSimRoot*/maize model for each nodal root. *RootSlice* scales to subcellular phenotypes such as vacuolar dimensions. Redrawn from Sidhu et al. (2023)

Several functional-structural models of root architecture including Archisimple, RootTyp, *OpenSimRoot* (and its forerunner *SimRoot*)(Fig. 19), ROOTMAP, SPACSYS, R-SWMS, RootBox, CRootBox have been used to study various aspects of root-soil interactions (Pages et al. 2004, 2014; Wu et al. 2007; Javaux et al. 2008; Postma et al. 2017; Leitner et al. 2010; Schnepf et al. 2018; Lynch et al. 1997; Diggle 1988; Moraes et al. 2019b, a; Dunbabin et al. 2013a, b; Postma and Black 2021). Root models have been successfully used to evaluate various architectural and anatomical phenotypes for nitrogen capture (Postma et al. 2014; Rangarajan et al. 2018; Rangarajan et al. 2022; Saengwilai et al. 2021; Perkins and Lynch 2021; Ajmera et al. 2022; Postma and Lynch 2011a, b; Schneider et al. 2017a, b; Schneider et al. 2020a, b), to identify optimal root architectures for nitrogen and water uptake (Dunbabin et al. 2003; Renton and Poot 2014; Ho et al. 2005, Rangarajan et al. 2022, Ajmera et al. 2022), inter- and intra-specific root competition (Postma and Lynch 2012; Dunbabin 2007; Hoffland et al. 1990), kinetics of nitrogen uptake (York et al. 2016a, b), and nitrogen capture under different soil physical scenarios (Strock et al. 2022a, b). Root anatomical phenotypes influence resource acquisition and several modelling frameworks exist which capture root anatomy including MECHA (Couvreur et al. 2018), OpenAlea (Pradal et al. 2008), GRANAR (Heymans et al. 2020), and *RootSlice* (Sidhu et al. 2023). *RootSlice* in particular allows accurate quantification of rhizoeconomic variables involving carbon, nitrogen and phosphorus (Fig. 19) (Sidhu et al. 2023).

Root models simulating nitrogen capture aim to capture processes occurring at different scales in the root system from µm (e.g., root anatomy and root hairs) to mm (e.g., root segments) to multiple cm (whole root system) while also simulating hydrologic and soil processes affecting the transformations and movement of nitrogen in the soil as well as processes occurring in the rhizosphere. A major setback of the resulting computationally demanding complexity is that on one hand 3D functional structural root models cannot simulate full crop cycles, while field-scale crop models are less computationally demanding yet represent root distribution through 1D vertical density profiles thus trivializing root phenotypes. The integration of root models with robust models of shoots, soil, microbes and agroecologies will comprise increasingly powerful tools towards developing crops and cropping systems, a requirement to sustainably provide for an increasing population in a degrading environment (Benes et al. 2020). Modeling and integrating processes across scales that are on different biological, temporal and computational scales however is a challenge (Baldazzi et al. 2012; Band et al. 2012; Postma and Black 2021). Recent studies have explored different methods to integrate models in efforts to fulfill inadequacies in model integration and multi-scale modeling (Mai et al. 2018; Lobet et al. 2014; Fang et al. 2019; Lang 2019; Ajmera et al. 2022; Seidel et al. 2022a, b; Wu et al. 2007; Marshall-Colon et al. 2017; Benes et al. 2020).

Nitrogen bioavailability is highly variable in time and space and depends on soil management. Climate change as well as agricultural management affects nitrogen availability in the soil by affecting rhizosphere processes such as mineralization. Adopting sustainable agricultural methods such as conservation agriculture increases the formation and persistence of biopores which provide low resistance pathways for solute movement affecting nitrogen distribution, as well as root growth. A well-adapted phenotype in a high-input system might not be as efficient in low-input systems (Sect. "Low-Input vs. High-Input Systems": Lynch et al. 2022). While deep roots are thought to be useful in general, the benefits of reaching deeper depth through accessing biopores is uncertain (Gao et al. 2016). In silico methods facilitate the study of whole plants in whole soils and provide a useful complement to existing literature which focuses on field-scale responses of crops or responses of individual root axes (Lynch et al. 2022). A particularly promising aspect of simulation models is their use to explore decision spaces that are too vast to explore empirically. A recent study by Rangarajan et al (2022) demonstrates the application of artificial intelligence, Multi-Objective evolutionary algorithms in particular, to identify root phenotypes under various environments providing promising avenues for developing more resilient, efficient crops in future climate scenarios.

Developments in machine learning aspects of artificial intelligence have enabled progress in high-throughput phenotyping and related advances aboveground. However, progress belowground in root related research has been limited due to the complexity, plasticity, inaccessibility as well as the presence of artifacts due to technicalities in acquiring and interpreting images obtained from an opaque medium, the soil (Rangarajan and Lynch 2021). In silico tools are valuable to bridge the gap in phenotyping by providing a virtual platform for high-throughput phenotyping of root phenotypes (Rangarajan and Lynch 2021; Burridge et al. 2020a, b). Studies that use machine learning to study root phenotypes are limited to seedling phenotyping (Falk et al. 2020), this limitation can be effectively overcome by using in silico approaches (Benes et al. 2020; Rangarajan and Lynch 2021). In silico phenotyping with methods that enable exploration of high dimensional decision spaces of the various soil and climatic environments can enable identification of root phenotypes that match the environment.

## Phene integration and multi-objective optimization for breeding strategies

### The value of ideotype breeding

The complexity of the fitness landscape for root phenotypes calls for informed selection of specific phenotypes for specific agroecologies, i.e. *ideotype breeding* (Donald 1968; Lynch 2019). The number of integrated phenotypes resulting from the interplay of many phenes and their interactions with the environment create an extremely large number of scenarios. For example, 6 root phenes each existing in only 3 states (*e.g.*, small, medium, large) generates 3^6^ (*i.e.* 729) integrated phenotypes, each of which may have significant interactions with nitrogen availability in diverse soils, climates, and management regimes. Brute-force yield selection is therefore highly unlikely to identify optimal root phenotypes that coincidentally exist in elite germplasm that also possesses local adaptation, vigor, disease resistance, etc*.* This may account for the fact that brute-force yield selection for nitrogen efficiency has generally been slow and costly. It is also probable that elite germplasm, usually selected under high-input conditions, may not possess root phenotypes conferring adaptation to infertile soil. Root ideotype breeding was successful in case of the topsoil foraging ideotype for phosphorus capture in legumes (Burridge et al. 2019). The S*teep, Cheap, and Deep* ideotype has been proposed for improved nitrogen capture in maize and other cereal crops (Lynch 2013). In the future, consideration of whole-plant phene integration must be coupled with co-optimization approaches in plant breeding rather than a singular focus on yield (York et al. 2022).

### Phenes are more useful than ‘traits’

A phene is an elemental unit of the phenotype at a given level of organization (Lynch and Brown 2012; York et al. 2013). As selection criteria, phenes are more useful than traits that aggregate multiple phenes (in the extreme case, yield itself), because phenes are axiomatically under simpler genetic control than any combination of phenes. Phene selection also permits informed assembly of an optimal phenotype. For example, root depth is an important trait for the capture of subsoil nitrogen in maize, but root depth aggregates multiple distinct phenes, including axial root growth angle, reduced production of crown roots, reduced lateral branching density, RCA formation, reduced cortical cell file number, and increased cortical cell size. These six phenes are under distinct genetic control (Schneider et al. 2020a, b; Schneider and Lynch 2020) and have important interactions with each other and with the soil environment. Selection for root depth in a breeding program will therefore be less informative and more complex, both genetically and physiologically, than would selection for specific combinations of specific phenes. As discussed here, phene integration and interactions must be considered (York et al. 2013). Research in phenomics has led to the combination of large genetic studies with crop physiology, providing new opportunities for knowledge creation. Functional phenomics has been proposed as a new field of inquiry allowed by large-scale measurements of numerous interacting phenes across diverse taxa that facilitate statistical analysis to infer how phenes relate to one another and to plant performance (York 2019). At the same time, functional phenomics applies simulation modeling as discussed in Sect. "Exploring the Fitness Landscape of the Root Phenome in silico" to both validate newly discovered phenes as well as to explore phenome space for the most promising phenes to target in phenotyping campaigns. Therefore, functional phenomics may address critical knowledge gaps to leverage physiological mechanisms in crop breeding.

### Phenotyping roots

An obvious bottleneck to the deployment of root phenotypes in crop breeding is the challenge of assessing the root phenotypes of a large number of plants in a meaningful, cost-effective way. In some cases, high-throughput phenotyping of seedlings grown in controlled environments may provide useful information. For example, root architecture of common bean seedlings grown in controlled environments is associated with yield performance in a large diversity panel grown in many diverse production environments (Strock et al. 2019a). Another important example is that of root hair length and density, which can be evaluated in young plants grown in germination ‘roll-ups’ (Vieira et al. 2007). Other examples exist, and several platforms have been developed for high-throughput phenotyping of root architecture in controlled environments (Atkinson et al. 2019), but such systems require validation in the target field environments, and suffer from difficulties associated with creating accessible yet realistic root growth environments that mimic key features of natural soil, as well as the challenge of managing root growth containers of sufficient size, since small pot size often restricts root development (Poorter et al. 2012, 2016). Furthermore, seedlings may not manifest meaningful aspects of the mature root phenotype, as is true for example with nodal roots in cereal crops, which provide the majority of water and nutrients to mature plants but which are not present in seedlings. In addition, anatomical phenotypes of roots emerging from older shoot nodes are distinct from those expressed in younger shoot nodes in maize (York and Lynch 2015; Yang et al. 2019).

For these reasons root phenotyping of mature plants in the field is an attractive option. In recent years several such platforms have been developed for both architectural and anatomical phenotypes. Phenotyping of root system architecture in the field is most often accomplished by excavating roots from soil followed by imaging. *Shovelomics*, or root crown phenotyping (Trachsel et al. 2011; Burridge et al. 2016), involves excavating the top portion of the root system, removing the soil, and photographing with a digital camera. Image analysis tools have been developed such as *DIRT* (Bucksch et al. 2014; Liu et al. 2021) and *REST* (Colombi et al. 2015). To streamline this process, the RhizoVision Crown hardware and software platform was developed that combines a backlight with a monochrome camera to capture root crown silhouettes that facilitate image analysis (Seethepalli et al. 2021). The *DIRT* platform has recently been extended to a 3D photogrammetry method with 3D volume analysis (Liu et al. 2021). Soil coring is another popular (albeit laborious, invasive, and noisy) phenotyping method that can complement shovelomics since it allows quantification of roots deeper in the soil profile. Soil coring typically involves removing a core of soil, dividing into vertical increments, washing and collecting the roots, and scanning roots on a flatbed scanner with a transparency unit. The ‘core break’ method permits estimation of root distribution with depth in the field without laborious core washing and quantification of clean roots (Wasson et al. 2016). Recent research has demonstrated that the positions of soil cores can influence the ability of the method to distinguish root system properties among genotypes, and to most accurately reflect field-level attributes (Burridge et al. 2020a). The free and open-source *RhizoVision Explorer* software has quickly been adopted by the root biology community as a replacement for *WinRhizo* due to its usability, speed, and accuracy (Seethepalli et al. 2021). Field phenotyping of root anatomy is possible by combining shovelomics with high-throughput laser ablation tomography followed by image analysis with *RootScan* or other tools (Strock et al. 2019b, 2022b; Lynch et al. 2021; Strock et al. 2022a, b). LEADER (Leaf Elemental Accumulation from Deep Roots) can estimate root depth of maize (and probably any other taxa) in the field from elemental analysis of leaves, which can be conducted with nondestructive means such as hand-held X-Ray Fluorescence Spectroscopy.

## Future prospects

Crops with reduced requirement for nitrogen fertilizer would make substantial contributions to a central challenge of the twenty-first century: how to assure food security for 10B people in a degraded global environment while mitigating climate change. As we show in this essay, a wide array of root phenotypes are excellent potential selection targets for the development of crops with superior nitrogen capture. For some of these, further research is warranted, while others are understood well enough to justify their deployment in breeding programs. In all cases substantial genotypic variation is present in crop germplasm. In some cases, high throughput phenotyping platforms are available, and in a few cases regulatory genetic loci have been identified.

This being the case, it is rather surprising that relatively little effort has been devoted to improving crop nitrogen capture by deploying superior root phenotypes. This is as true in rich nations, which would benefit from reduced production costs and environment impacts resulting from reduced nitrogen fertilization, as it is in developing nations, in which such crops would improve crop production, farm income, and food security. There are several possible reasons for this. One is the complexity of nitrogen capture by roots, since both root phenotypes and soil nitrogen availability are spatiotemporally complex and can be influenced by management regimes and soil taxa. Expertise in these topics is rare and is not typically represented in breeding programs. Several of the concepts, tools and paradigms presented in this article are relatively novel, which exacerbates the scarcity of relevant expertise. For example, several of the phenotypes discussed here have very few literature citations, and modern in silico tools for multiscale mechanistic modeling have scant engagement in the plant research community, despite the growing importance of computational biology, which is destined to become an indispensable tool in coming decades.

We stand on the verge of a nascent paradigm shift in plant biology, from a focus on the genome to a broader focus on the phenome as a whole and how it regulates adaptation to diverse environments. Understanding the fitness landscape of root phenotypes for improved nitrogen capture requires integration across scales and across disciplinary siloes. Transdisciplinary teams are needed, as is greater attention to the agroecosystems of developing nations, which are underserved by research efforts in rich nations. A ‘whole plant in whole soil’ approach (Lynch et al. 2022), emphasizing actual crops in actual field soil is needed, rather than model organisms in artificial growth media. The substantial benefits for food security, agricultural sustainability, and climate amelioration make reduced nitrogen demand an essential element of the more resilient and productive crops and cropping systems urgently needed in global agriculture.

## Dedication

We dedicate this article to Emmanuel Epstein, who passed last year at age 106 after a lifetime of service and many seminal contributions to the field of plant nutrition.

## Abbreviations

BNF: Biological Nitrogen Fixation
CCS: Cortical Cell Size
CCFN: Cortical Cell File Number
LCA: Living Cortical Area
MCS: Multiseriate Cortical Sclerenchyma
RCA: Root cortical aerenchyma
RCS: Root cortical senescence
